# Acoustic vibrational resonance in a Rayleigh-Plesset bubble oscillator

**DOI:** 10.1016/j.ultsonch.2020.105346

**Published:** 2020-09-23

**Authors:** K.A. Omoteso, T.O. Roy-Layinde, J.A. Laoye, U.E. Vincent, P.V.E. McClintock

**Affiliations:** a Department of Physics, Olabisi Onabanjo University, Ago-Iwoye, Ogun State, Nigeria; b Department of Physical Sciences, Redeemer’s University, P.M.B. 230, Ede, Nigeria; c Department of Physics, Lancaster University, Lancaster LA1 4YB, United Kingdom

**Keywords:** Bubble oscillator, Vibrations, Fluctuations, Resonance, Acoustic waves

## Abstract

The phenomenon of vibrational resonance (VR) has been investigated in a Rayleigh-Plesset oscillator for a gas bubble oscillating in an incompressible liquid while driven by a dual-frequency force consisting of high-frequency, amplitude-modulated, weak, acoustic waves. The complex equation of the Rayleigh-Plesset bubble oscillator model was expressed as the dynamics of a classical particle in a potential well of the Liénard type, thus allowing us to use both numerical and analytic approaches to investigate the occurrence of VR. We provide clear evidence that an acoustically-driven bubble oscillates in a time-dependent single or double-well potential whose properties are determined by the density of the liquid and its surface tension. We show both theoretically and numerically that, besides the VR effect facilitated by the variation of the parameters on which the high-frequency depends, amplitude modulation, the properties of the liquid in which the gas bubble oscillates contribute significantly to the occurrence of VR. In addition, we discuss the observation of multiple resonances and their origin for the double-well case, as well as their connection to the low frequency, weak, acoustic force field.

## Highlights

• Vibrational resonance (VR) is reported in a Rayleigh-Plesset gas bubble model.
• The complex dynamics is described by a classical particle in a time-dependent single or double-well potential.
• Single and multiple VR can occur by the variation of the parameters of the acoustic waves and the properties of the liquid.

## Nomenclature

**Roman letters**
$A_{c}$: Amplitude of the cosine component of the output signal
$A_{L}$: Linearized response amplitude from equilibrium
$A_{s}$: Amplitude of the sine component of the output signal
*c*: Speed of light (m/s)
$C_{i}$: Derived constants of derived equation
f: Amplitude of the fast driving field
*F*: Amplitude of solution of linearized deviation
$F_{\mathrm{acc}}$: Weak acoustic driving force
HIFU: High Intensity Focused Ultrasound
*n*: Number of complete oscillations
ODE: Ordinary Differential Equation
$P_{\mathrm{ext}}$: External pressure on the bubble
$P_{\mathrm{in}}$: Pressure in the bubble
$P_{0}$: Ambient pressure (atm)
$p_{\mathrm{st}}$: Acoustic pressure
*Q*: Response amplitude
*R*: Instantaneous bubble radius (m)
$\overset{¨}{R}$: Second time derivative of *R*
$R_{0}$: Equilibrium bubble radius (m)
SR: Stochastic resonance
*t*: Time (s)
*T*: Period of bubble oscillation (s)
$V_{\mathrm{eff}}(\chi )$: System’s effective potential
VR: Vibrational resonance

**Symbols**
V(x): System’s potential
x(t): Dimensionless instantaneous bubble radius
$\overset{¨}{x}$: Second time derivative of x(t)
Y(t): Deviation of the slow motion

**Greek letters**
$\alpha_{i}$: Coefficients of nonlinear damping
β: Square of system’s resonance frequency
γ: Coefficient of potential nonlinearities
δ: Effective contributions to $P_{0}$
∊: Amplitude of weak acoustic excitation
η: Coefficient of the quadratic damping
κ: Polytropic exponent
λ: Coefficient of highest nonlinearity
$\mu_{l}$: Viscosity of the liquid (Pas)
$\mu_{\mathrm{th}}$: Effective thermal viscosity (Pas)
ρ: Density of the liquid ($\mathrm{kg}/m^{3}$)
σ: Surface tension coefficient (N/m)
τ: Periodic fast time
ϕ: Phase angle
χ(t): Component of slow oscillation with ω
ψ(t): Component of fast oscillation with Ω
ω: Frequency of weak acoustic excitation
$\omega_{r}$: Resonance frequency
Ω: Frequency of the fast acoustic field
overdot: Time derivative
$\overset{‾}{\psi }$: Time average of ψ
〈〉: Time average

## 1 Introduction

Nonlinear dynamical systems are ubiquitous in nature and they exhibit a diversity of fascinating phenomena. One of these is *resonance*, which may occur in both linear and nonlinear dynamical systems [1], [2], [3], [4]. The analysis of resonance, especially in nonlinear systems, has received close attention in the scientific community, both pure and applied. The fundamental understanding of resonance is highly relevant to numerous practical applications in many branches of science, engineering, and medicine [1], [5], [6], [7], [8]. In general, *resonance* implies the matching of two or more frequencies within a system and, in most dynamical systems, it can appear in various forms when the frequency of an external driving force is matched to the natural frequency of the system, when it gives rise to an enhancement of the system’s response [1], [8], [9], [10]. The type of resonance depends largely on the matching method as well as on the nature of the external driving force that produces it [11], [12]. Vibrational resonance, for example, occurs in dual-frequency-driven non-linear systems with distinct frequencies [7], [8], [9], [10], [13], [14], [15], [16], [17], [18], [19]; stochastic resonance (SR) occurs when one of the two forces is replaced with noise of appropriate intensity [20], [21], [22], [23], [24], [25], [26], [27], [28]; and chaotic resonance takes place when the high-frequency component of the driving force is replaced with the output from a chaotic system [29], [30], [31]. Other forms of resonance include coherence resonance [32], [33], [34], ghost resonance [21], [35], [36] and autoresonance[37], [38], parametric resonance [39], [40], [41], as well as conventional resonance in the form of harmonic, subharmonic and ultraharmonic resonances [42], [43], [44].

Our focus here is on the phenomenon of vibrational resonance (VR), originally proposed by Landa and McClintock [9]. In VR, a nonlinear system driven by a low-frequency signal is also subjected to a second, but high-frequency harmonic excitation, referred to as the *fast-signal*, such that the frequency (Ω) of the high-frequency component is significantly greater than the frequency (ω) of the low-frequency component. Under these conditions, the response amplitude of the system at the frequency of the low-frequency component versus the amplitude of the fast signal exhibits an “inverted-U-shaped” curve, similar to the signal-to-noise ratio curve observed in the SR phenomenon. By tuning the amplitude of the auxiliary signal or other parameter of the system, the weak/low-frequency excitation can be greatly amplified by VR. Basically, the fast signal acts as a control input to the system through the adjustments of its amplitude, frequency and phase (See Ref. [1] and references cited therein for further details). The phenomenon has gained enormous research attention in the last two decades and has been extensively investigated due its several potential industrial and biomedical applications in a wide range of contexts including bistable systems [45], [46], [47], [48], multistable systems [19], [49], systems with various forms of potential structures [13], [15], linearly damped systems [1], [50], electrical circuits and time-delayed systems [5], [6], [51], [52], as well as plasma models [10], [53]. Although earlier VR studies focused on the parameters of the high-frequency component of the bi-harmonic force, some recent VR research has explored the effects of parameters such as nonlinear damping/dissipation [7], [10], [53], [54], the complementary roles played by system’s innate bifurcation parameters [13], [48], [55], the effect of time-delay [52], [56] and fractional derivatives [57], [58], [59], antiresonance [60], the depth of a potential and the location of its minimum[61], the effects of potential deformation [62] and potential roughness [53], and mass-dependent VR [63], amongst others. More importantly, VR has been realized experimentally in vertical cavity surface emitting lasers and optical systems [45], [46], [49], [64], [65]. The aforementioned investigations have enriched our understanding of the VR phenomenon, its associated mechanism, and the roles played by relevant system parameters in inducing or promoting VR, as well as in suggesting potential applications.

Some intriguing properties, novel phenomena and potential applications have been explored and reported. For instance, Gandhimathi et al. [66] examined the relationship between SR and VR, while Morfu and Bordet [67] recently established a connection between phase-locking modes and VR. Rajamani et al. [68] observed a novel ghost-vibrational resonance phenomena in a system driven by multi-frequency signals and extended their analysis to a network of oscillators. A novel vibrational antiresonance in which a strong signal-suppression occurred due to a large negative phase-shift in coupled oscillators was very recently reported by Sarkar and Ray [60]. Furthermore, the existence of subharmonic and superharmonic resonances [69] and higher harmonics [70] have also been reported. In a different sequence of developments, new methods of analysis in which nonlinear systems are driven by *aperiodic* forces, in contrast to periodic driving, have been investigated and has given birth to what is termed *aperiodic vibrational resonance*. There are a number of its variants, including re-scaled aperiodic vibrational resonance and the double-sampling aperiodic vibrational resonance methods [71], [72]. In a very recent paper, a new approach to VR in terms of a spectral amplification factor was presented [73]. All these results are directed towards exploring the diverse potential applications of VR in, for instance, improving energy harvesting from electromechanical vibration devices [74], [75], [76] as well as in enhancing generalized energy detector approaches to weak random signals detection [77]. Another important application is concerned with signal processing. In this direction, VR has been proposed for the transmission, filtering and amplification of signals; such applications have been demonstrated both theoretically and experimentally [78], [79], [80], [81]. In rotational machinery quite generally, bearings play crucial roles e.g. in mining, aerospace, railways, and metallurgy. When they become faulty, the equipment typically fails, sometimes catastrophically. In this context, VR has been proposed as a promising technique for the early noninvasive diagnosis of nascent bearing faults [82], [83], [84], [85], [86].

In the light of the forgoing, we investigate and analyze the VR phenomenon in a model of an amplitude-modulated, acoustically-driven gas bubble in an incompressible liquid which, to the best of our knowledge, has not previously been discussed. Bubbles appear in both the life sciences and natural sciences, as well as in technology [43], and their dynamics has received considerable attention in connection with acoustic cavitation [42], [43], [44] where bubbles are excited by an acoustic sound field mostly consisting of single or dual frequencies. In addition to the existence of bifurcation structures and chaotic behaviour, bubble dynamics is rich in special classes of resonance, and these have constituted one important focus of recent research. For instance, the nonlinear dynamics of bubbles under dual-frequency acoustic excitation was numerically investigated recently within a wide range of parameters by Zhang et al. [44]. Two significant resonance features, namely, combination resonance and simultaneous resonance were observed. Moreover, parameters such as the bubble radius, the acoustic pressure amplitude, and the energy distribution between wave components were reported to be of crucial importance in that they impacted significantly on the nonlinear bubble oscillation. Under multi-frequency acoustic cavitation, the dynamics of bubble oscillations also shows evidence of resonances [44]. However, despite the huge number of investigations of bubble oscillations, and the wide range of potential applications of multi-frequency excitations of gas bubbles, the dynamics of gas bubbles driven by dual-frequency forces is yet to be fully investigated and understood. In sonochemistry, fluid engineering and medicine (in particular, medical diagnostics and therapy), where acoustic cavitation due to bubble formation is well-known, a multi-frequency acoustic sound field promotes system effectiveness and can also be useful in controlling the chaotic dynamics of the bubble oscillations, especially where they are undesirable [2], [87], [88]. In addition, the dual-frequency approach is employed in designing sonochemical reactors [89], synthesis with nanoparticles [90] and sonoluminescence [91], and for investigating bubble size distributions with void fraction in the open ocean [92], in studying the dynamic variations of bubble radii during oscillations [93], [94], and in the enhancement of the ultrasound accuracy of biomedical diagnosis [95], [96], and the efficiency of tumor therapy, including tumor ablation [97]. The dual-frequency approach can also accelerate bubble growth through mass transfer, leading to the generation of large bubbles [44], [96], [98]. We draw attention to a very recent study of dual-frequency-driven bubbles in a high-dimensional parameter space based on GPU accelerated computations [99]. It represents the state-of-the art in applications of dual-frequency irradiation and also provides an extensive review ranging from increased sonochemical yields to decreased sonochemical efficiency due to the synergetic effects of dual-frequency driving.

Consequently, there is a compelling need for further investigation of the nonlinear responses of dual-frequency driven oscillators to the effects of high-frequency components in the context of the VR of gas bubbles driven by an amplitude-modulated acoustic field. We undertake such an investigation here. In addition to the parameters of the modulating force, and on account of the multi-parametric nature of the bubble oscillator, the possible contributions of the parameters of the bubble and its surrounding medium (in this case liquid) to the occurrence of VR are investigated as they can in principle provide information essential for addressing other problems arising in acoustic cavitation. We provide numerical evidence for the occurrence of VR as well as for its enhancement by amplitude modulation over a wide parameter range. Our results are validated theoretically based on the method of separation of time-scales. The rest of the paper is organized as follows. In the following section (Section 2), we present the Rayleigh-Plesset bubble oscillator model – expressing the complex equation as the dynamics of a classical particle in a potential well of the Liénard type [100] for the first time. We provide evidence that, using the Rayleigh-Plesset bubble model, an acoustically-driven bubble oscillates in a time-dependent single or double-well potential whose properties are determined by the density of liquid and its surface tension. Section 3analyzes and discusses the stability of equilibrium points. In Section 4, detailed analytical treatments of the resonance behaviour are given. Numerical simulation results, complemented by theoretically computed results are presented in Section 5. The paper is concluded with the identification of some highlights, in Section 6.

## 2 The model

The model of interest is the Rayleigh-Plesset equation for small-amplitude oscillations of a spherical gas bubble in an infinite incompressible liquid [101]. In the model equation, the bubble is treated as an oscillatory system (bubble oscillator) that incorporates various parameters of the surrounding liquid. In general, the size of the bubble and its gas/vapour contents are specified. Thus, for a spherical gas bubble, the defining variables for its size and shape can be consolidated into the time-dependent bubble radius R(t). In addition to R(t), other essential parameters include the equilibrium radius ($R_{0}$), the external pressure on the liquid ($P_{\mathrm{ext}}$), and the bubble’s internal pressure $P_{\mathrm{in}}$, as well as the polytropic exponent of the gas in the bubble (κ), the density of the liquid (ρ), the liquid and thermal viscosities ($\mu_{l},\;\mathrm{and}\;\mu_{\mathrm{th}}$), and the surface tension (σ) of the liquid.

When driven by acoustic fields, the bubble dynamics may be likened to that of a damped-driven oscillator and, during the bubble oscillatory motion, the contained interior gas behaves polytropically. The polytropic exponent κ describes the thermodynamics state of the gas in the bubble. It depends on the acoustic frequency of the driving field, the bubble radius, and the nature of the gas. In general, if the bubble is sufficiently large or the driving frequency is sufficiently high, the bubble will behave adiabatically because there will be a significant temperature gradient across its radius [102]. In this case, κ≈ the ratio of specific heats. If, on the other hand, the bubble is sufficiently small or the driving frequency is sufficiently low the bubble will behave isothermally. Intermediate thermodynamic states between these two extremes have been identified [102]. In principle, the VR phenomenon requires a very high-frequency driving force. It is therefore appropriate to assume the adiabatic state, so that κ=1.3.

Moreover, it has long been known that the natural oscillations of a bubble are dependent on the effective damping mechanisms arising from the liquid and thermal viscosities. There is also a viscosity due to acoustic radiation to be considered, as well as damping due to mass diffusion and to the evaporation–condensation of vapour [103]. In experimental studies, however, damping due to mass diffusion and to the evaporation–condensation of vapour are often neglected [102]. While the other sources of damping are clearly understood, the origin of thermal viscosity is not straightforward. Unlike purely isothermal processes in which pressure and volume changes are mutually in phase, a phase difference exists in real bubbles and the gas can behave adiabatically. The latter processes are associated with a net flow of heat energy into the liquid when work is done on the gas, reducing its volume by increasing the pressure during compression. This work done on the gas is far greater than the work done by the gas in moving the surrounding liquid during expansion, thereby increasing the internal energy of the gas as well as transferring energy by conduction through the gas into the surrounding liquid. The net flow of heat energy is characterised by a thermal viscosity that has been reported to influence the free oscillations of the bubble strongly, particularly during the transition between the adiabatic and the isothermal states [103], [104], [105]. Recently, the impact of non-linear damping/dissipation on the occurrence of VR in nonlinear systems has also gained considerable research attention [7], [10], [53], [54]. In view of this, and to account for the roles of all the individual factors contributing to damping of the bubble dynamics, and their influence on the VR phenomenon, it is essential to treat the liquid and thermal viscosities, as well as the acoustic radiation, independently in the derivation of the equation of motion. This contrasts with the traditional approach of defining an effective viscosity whose effective contributions may appear negligible in the VR analysis.

Here, we consider the Rayleigh-Plesset equation for bubble oscillations which can be written [43], [101], [106], [107], [108];

(1) $$R\overset{¨}{R}+\frac{3}{2}{\overset{˙}{R}}^{2}=\frac{1}{\rho }[P_{\mathrm{ext}}(\overset{˙}{R},R,t)-F_{\mathrm{acc}}(t)],$$

where

(2) $$P_{\mathrm{ext}}(\overset{˙}{R},R,t)=P_{\mathrm{in}}-\frac{2\sigma }{R}-\frac{4\mu_{l}}{R}\overset{˙}{R}$$

and

(3) $$F_{\mathrm{acc}}(t)=P_{0}[1+∊\cos\omega t]\text{.}$$

Here $P_{0}$ is the ambient pressure, and ∊ and ω are the amplitude and angular frequency of the external acoustic excitation. To facilitate theoretical and numerical investigation of VR, it is useful to express Eq. (1) as the classical dynamics/motion of a particle in a potential well. This enables us to elucidate the nature of the potential within which a bubble oscillates – an important issue in the study of dynamical systems that was overlooked in previous research on bubble dynamics. Thus we will, for the first time, obtain an exact expression for the potential well in which the bubble oscillates in the context of the Rayleigh-Plesset bubble model. We will follow the steps in [109], expressing the bubble radius *R*, in terms of a dimensionless variable *x*, i.e. $R=R_{0}(1+x)$, and write $P_{\mathrm{in}}$ as

(4) $$P_{\mathrm{in}}=P_{\mathrm{in},\mathrm{eq}}+P_{0}p(\overset{˙}{R},R,t),$$

where

(5) $$\begin{array}{cc} P_{\mathrm{in},\mathrm{eq}}= & P_{0}+\frac{2\sigma }{R_{0}},\\ P_{0}p(\overset{˙}{R},R,t)= & P_{\mathrm{in},\mathrm{eq}}\left[{\left(\frac{R_{0}}{R}\right)}^{3\kappa }-1\right]-4\mu_{\mathrm{th}}\frac{\overset{˙}{R}}{R}\text{.} \end{array}$$

Eq. (2) is the continuity condition for the surface tension across the bubble interface, governing its forced radial oscillations [103]. $P_{\mathrm{ext}}(\overset{˙}{R},R,t)$ is the pressure at the bubble wall in the liquid satisfying the continuity condition, and $P_{0}p(\overset{˙}{R},R,t)$ is the dimensionless deviation from the equilibrium pressure. For small-amplitude oscillations one considers thermal effects in the bubble content leading to strong damping on the transitional behavior of the bubble. At Blake’s critical threshold [110], the equilibrium radius of the bubble depends only on this tension and, assuming the equilibrium radius of the bubble is in the neighborhood of the critical radius, the derivative of the tension with respect to the equilibrium radius of the bubble can be approximated as

(6) $$\begin{array}{cc} P_{0}p(\overset{˙}{R},R,t)= & P_{\mathrm{in},\mathrm{eq}}\left[{\left(\frac{R_{0}}{R}\right)}^{3\kappa }-1\right]-4\mu_{\mathrm{th}}\overset{˙}{x}\\ & \approx -3\kappa P_{\mathrm{in},\mathrm{eq}}x-4\mu_{\mathrm{th}}\overset{˙}{x} \end{array}$$

in dimensionless form [103], [111], [112]. With $F_{\mathrm{acc}}(t)=P_{0}[1+∊\cos\omega t]$, and substituting Eqs. (2), (3), (4), (5), (6) into Eq. (1), the Rayleigh-Plesset Eq. (1) then becomes

(7) $$R_{0}\overset{¨}{x}+\frac{3R_{0}\overset{˙}{x^{2}}}{2(1+x)}=\frac{1}{\rho R_{0}(1+x)}P_{\mathrm{in},x}\text{.}$$

where $P_{\mathrm{in},x}=[P_{\mathrm{in},\mathrm{eq}}-3\kappa P_{\mathrm{in},\mathrm{eq}}x-4\mu_{\mathrm{th}}\overset{˙}{x}-\frac{2\sigma }{R_{0}(1+x)}-\frac{4\mu_{l}\overset{˙}{x}}{\left(1+x\right)}-F_{\mathrm{acc}}]$. Using a binomial expansion, we can express the multiplicative term ${\left(1+x\right)}^{-1}$ as ${\left(1+x\right)}^{-1}=(1-x+x^{2}+\ldots )$: retaining up-to the quadratic term. After some algebraic manipulation, it is easy to show that Eq. (7) can be written as:

(8) $$\begin{array}{cc} \overset{¨}{x}+ & \frac{3}{2}\overset{˙}{x^{2}}(1-x+x^{2})+\frac{4\overset{˙}{x}}{\rho R_{0}^{2}}[(\mu_{\mathrm{th}}+\mu_{l})-x(\mu_{\mathrm{th}}+2\mu_{l})\\ & +x^{2}(\mu_{\mathrm{th}}+3\mu_{l})-2\mu_{l}x^{3}+\mu_{l}x^{4}]\\ & =\frac{x}{\rho R_{0}^{2}}\left[\frac{4\sigma }{R_{0}}-P_{\mathrm{in},\mathrm{eq}}(1+3\kappa )+F_{\mathrm{acc}}\right]\\ & +\frac{x^{2}}{\rho R_{0}^{2}}\left[P_{\mathrm{in},\mathrm{eq}}(1+3\kappa )-\frac{6\sigma }{R_{0}}-F_{\mathrm{acc}}\right]\\ & +\frac{x^{3}}{\rho R_{0}^{2}}\left[\frac{4\sigma }{R_{0}}-3\kappa P_{\mathrm{in},\mathrm{eq}}\right]-\frac{2\sigma x^{4}}{\rho R_{0}^{3}}\\ & +\frac{1}{\rho R_{0}^{2}}\left[P_{\mathrm{in},\mathrm{eq}}-\frac{2\sigma }{R_{0}}-F_{\mathrm{acc}}\right]\text{.} \end{array}$$

Substituting for $P_{\mathrm{in},\mathrm{eq}}$ in Eq. (4) into Eq. (8) and using the definition of $F_{\mathrm{acc}}$ yields

(9) $$\begin{array}{cc} \overset{¨}{x}+ & \frac{3}{2}\overset{˙}{x^{2}}(1-x+x^{2})+\frac{4\overset{˙}{x}}{\rho R_{0}^{2}}[(\mu_{\mathrm{th}}+\mu_{l})-x(\mu_{\mathrm{th}}+2\mu_{l})\\ & +x^{2}(\mu_{\mathrm{th}}+3\mu_{l})-2\mu_{l}x^{3}+\mu_{l}x^{4}]\\ & =\frac{x}{\rho R_{0}^{2}}\left[\frac{2\sigma }{R_{0}}(1-3\kappa )-3\kappa P_{0}+P_{0}∊\cos\omega t\right]\\ & -\frac{x^{2}}{\rho R_{0}^{2}}\left[\frac{2\sigma }{R_{0}}(2-3\kappa )-3\kappa P_{0}+P_{0}∊\cos\omega t\right]\\ & +\frac{x^{3}}{\rho R_{0}^{2}}\left[\frac{2\sigma }{R_{0}}(2-3\kappa )-3\kappa P_{0}\right]-\frac{2\sigma x^{4}}{\rho R_{0}^{3}}-\frac{P_{0}}{\rho R_{0}^{2}}∊\cos\omega t \end{array}$$

such that the Rayleigh-Plesset equation in Eq. (1) can be written in dimensionless form as

(10) $$\begin{array}{cc} \overset{¨}{x}+ & \overset{˙}{x}[\alpha_{0}-\alpha_{1}x+\alpha_{2}x^{2}-\alpha_{3}x^{3}+\alpha_{4}x^{4}]\\ & +\frac{3{\overset{˙}{x}}^{2}}{2}(1-x+x^{2})-x(\beta -\delta ∊\cos\omega t)\\ & +x^{2}(\gamma -\delta ∊\cos\omega t)-\gamma x^{3}+\lambda x^{4}=\delta ∊\cos\omega t, \end{array}$$

where the dimensionless parameters are

(11) $$\begin{array}{cc} \alpha_{0}= & \frac{4(\mu_{\mathrm{th}}+\mu_{l})}{\rho R_{0}^{2}},\alpha_{1}=\frac{4(\mu_{\mathrm{th}}+2\mu_{l})}{\rho R_{0}^{2}},\alpha_{2}=\frac{4(\mu_{\mathrm{th}}+3\mu_{l})}{\rho R_{0}^{2}},\\ \alpha_{3}= & \frac{8\mu_{l}}{\rho R_{0}^{2}},\;\alpha_{4}=\frac{4\mu_{l}}{\rho R_{0}^{2}},\;\beta =\frac{1}{\rho R_{0}^{2}}[\frac{2\sigma }{R_{0}}(1-3\kappa )-3\kappa P_{0}],\\ \delta = & -\frac{P_{0}}{\rho R_{0}^{2}},\lambda =\frac{2\sigma }{\rho R_{0}^{3}},\gamma =\frac{1}{\rho R_{0}^{2}}[\frac{2\sigma }{R_{0}}(2-3\kappa )-3\kappa P_{0}]\text{.} \end{array}$$

The equilibrium radius of the bubble, $R_{0}$ may take on values ranging from a micron or less (i.e. nanobubbles and microbubbles) up to several millimetres [42], [43], [111], [113], [114]. For underwater sparks or with explosives, the radius of the bubble may reach even larger values [43]. The dimensionless bubble parameter are given in Table 1 for the equilibrium radius $R_{0}=0.01$ m, and with the following values of the parameters: κ=1.3;σ=0.0725 Nm^−1^; $\mu_{\mathrm{th}}=0.001$ Pa s; ρ=998 kgm^−1^; $\mu_{l}=0.001$ Pa s; and $P_{0}=1$ atm.

**Table 1:** Calculated values of the system’s dimensionless parameters for an equilibrium radius $R_{0}=10$ mm. The values of the constants used are given in the text.

**S/N**	**Parameter**	**Value**
1	$\alpha_{0}$	0.0801
2	$\alpha_{1}$	0.1202
3	$\alpha_{2}$	0.1603
4	$\alpha_{3}$	0.801
5	$\alpha_{4}$	0.0400
6	β	−474.4
7	δ	−10.02
8	γ	-329.11
9	λ	145.38

Finally, we express Eq. (10) in terms of the bubble’s potential:

(12) $$\begin{array}{cc} \overset{¨}{x}+ & \overset{˙}{x}[\alpha_{0}-\alpha_{1}x+\alpha_{2}x^{2}-\alpha_{3}x^{3}+\alpha_{4}x^{4}]\\ & +\frac{3{\overset{˙}{x}}^{2}}{2}(1-x+x^{2})+\frac{\mathrm{dV}(x,t)}{\mathrm{dx}}=\delta ∊\cos\omega t, \end{array}$$

where V(x) is the bubble’s potential

(13) $$\begin{array}{cc} V(x,t)=-\frac{(\beta -\delta ∊\cos\omega t)x^{2}}{2}+ & \frac{(\gamma -\delta ∊\cos\omega t)x^{3}}{3}\\ & -\frac{\gamma x^{4}}{4}+\frac{\lambda x^{5}}{5}\text{.} \end{array}$$

We find that the Rayleigh bubble oscillator is a strongly nonlinear, parametrically excited, system characterized by higher-order nonlinear space-dependent dissipation of the Liénard type III [100], typical of fluidic plasma oscillators [115]. Parametric excitation arises from the modulation of the system’s potential when irradiated by acoustic fields, δ∊cosωt of amplitude δ∊, so that the potential V(x,t) is time-dependent. Note that previous works linearised the Rayleigh-Plesset equation (Eq. (1)))) to that of a harmonic oscillator by neglecting all powers of *x* and of its derivatives higher than the first (see e.g. Refs. [102], [103], [104], [112]). The corresponding truncation of higher-order nonlinearities in the potential and the damping functions impacted significantly on the nature and number of equilibrium points.

In the absence of the external acoustic field, the bubble would oscillate freely in the time-independent potential,

(14) $$V(x)=-\frac{\beta x^{2}}{2}+\frac{\gamma x^{3}}{3}-\frac{\gamma x^{4}}{4}+\frac{\lambda x^{5}}{5}\text{.}$$

The shape of the potential V(x) given by Eq. (14) describes the equilibria of the system and depends on the signs and values of the three dimensionless parameters, β,γ and λ as we will show in the following analysis. (See the Appendix I for mathematical details).

## 3 Equilibrium and stability

**Fig. 1:**
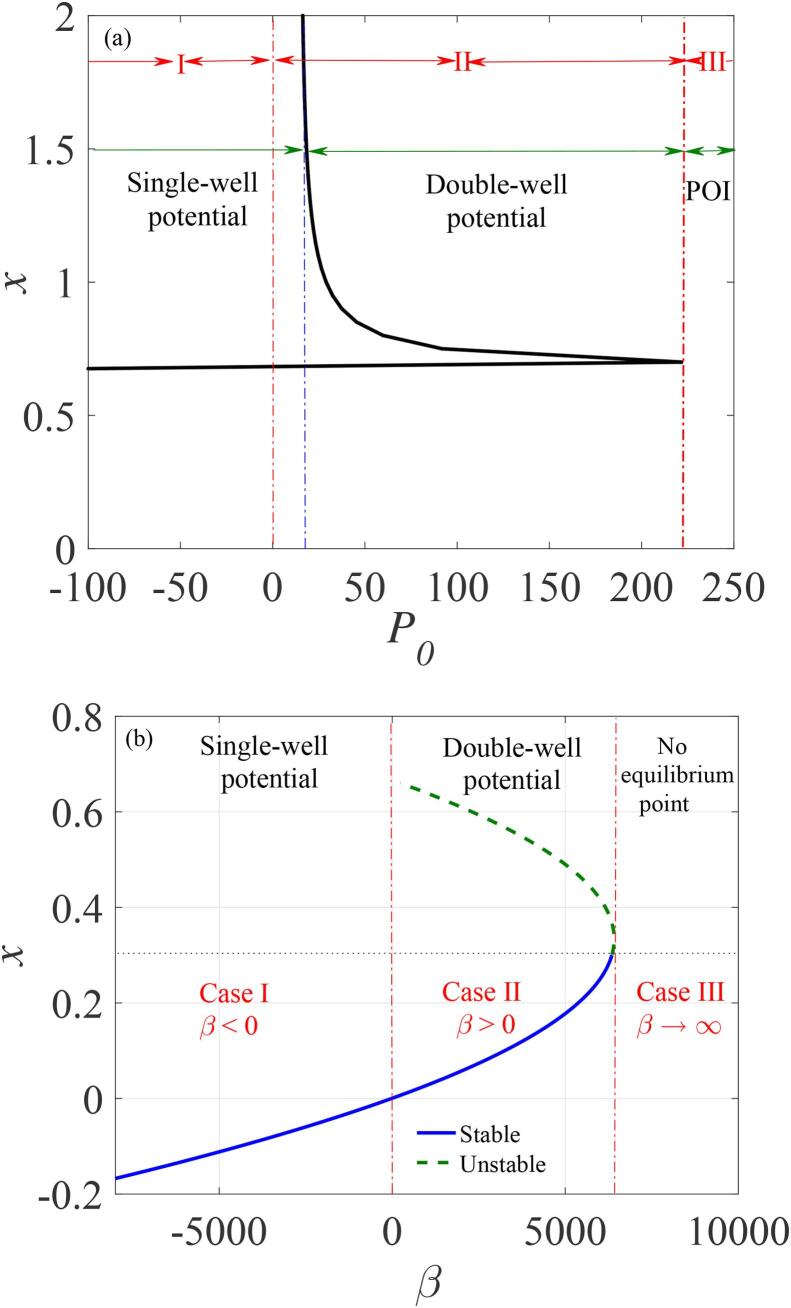
The dependence of the non-dimensional bubble radius *x* on; (a) the ambient pressure $P_{0}$ and (b) β the resonance frequency of the system.

To analyze the potential structure, we first revisit briefly the well-known theory of bubbles dynamics in relation to our model as shown in Fig. 1(a) [116], [117], [118], [119], [120], [121]. Accordingly, the oscillation of gas bubbles can be classified into three distinct regions relative to the special threshold which we summarise below.

(I) If the ambient pressure is higher than the vapour pressure of the liquid, the freely oscillating bubble posses only one stable equilibrium state, denoted as *case I* in Ref. [120].
(II) When the ambient pressure falls below the so-called Blake’s critical threshold or is in its neighbourhood, there exist two equilibria, one of which is stable while the other is unstable. This condition was classified as *case II* in Ref. [120].
(III) The trajectory of the system could tend to infinity whenever the ambient pressure is lower than the Blake’s critical threshold so that the dynamics is neither stable nor unstable, and without any local/global maxima/minima. This condition was classified as *case III* in Ref. [120].

Remarkably, *case II* and *case III* are separated by the Blake’s critical threshold which is a special limit that determines the stability boundary of the bubble system and also specifies the limit where bubbles tend to grow infinitely. Given the approximations employed in our model, our results can be considered to be in agreement with the existing literature [116], [117], [118], [119], [120], [121].

Using our proposed bubble oscillator model, we plot the dependence of the dimensionless bubble radius *x* on the dimensionless potential parameter β which controls the potential structure as shown in Fig. 1(b). It is obvious that the three distinct regions enumerated above are predicted by our model in a similar manner to the way in which the ambient pressure was used in previous works. Thus, one of the novelty of our model is its ability to employ the critical ambient pressure ($P_{0}^{\mathrm{cr}}=\frac{2\sigma (1+3\kappa )}{3\kappa R_{0}}$) estimated at the Blake’s threshold by equating λ=γ from Eq. (11) in predicting the potential well conditions, thereby providing an alternative approach to the exploration of bubble dynamics. Notably, the value of $P_{0}^{\mathrm{cr}}$ depends on the liquid surface tension σ which can be appropriately controlled [120], the equilibrium bubble radius ($R_{0}$) and the thermodynamic process defined by the polytropic parameter κ.

If the ambient pressure $P_{0}$ is greater than the estimated critical pressure ($P_{0}>P_{0}^{\mathrm{cr}}$), the dynamics of the system satisfies *case I* corresponding to a single-well potential condition for β<0. Furthermore, depending on the values of $P_{0}$, there is a narrow regime where *case I* (for β<0) overlaps with *case II* resulting in the coexistence of single-well (*case I*) and double-well (*case II*) oscillations which suggest the existence of multistability of attractors recently reported in coupled bubbles [122]. In addition, the dynamics when β=0 or slightly greater than zero does not translates to the dynamics for $P_{0}=0$. This is obvious in Fig. 1(a), and considering that γ is also dependent on the ambient pressure $P_{0}$. The double-well condition (β>0) which we termed case II arises if $P_{0}<P_{0}^{\mathrm{cr}}$, in agreement with the existing literatures [116], [120]. Finally, when β becomes extremely large such that β→∞, every trajectory of the system tends to infinity, and the *case III*, in which there is no equilibrium state is satisfied [116], [120].

**Fig. 2:**
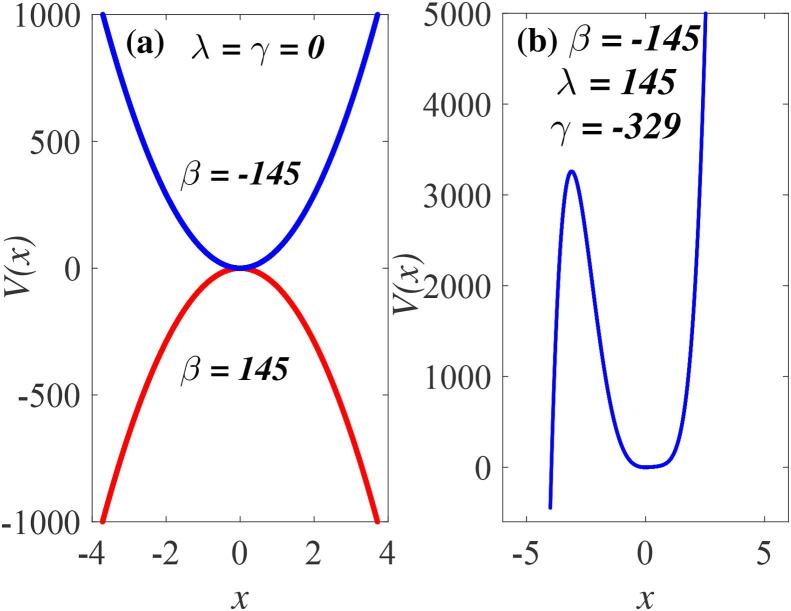
The bubble oscillator potential V(x) from Eq. (14) for single well conditions: (a) single-well for β<0,γ=λ=0 (a stable equilibrium) and single-hump for β>0,γ=λ=0 (an unstable equilibrium); (b) single-well-single-hump for β<0,γ<0 and λ>0, (two equilibria, one unstable and one stable equilibrium). In (b) the parameters are λ=145 and γ=-329.

**Fig. 3:**
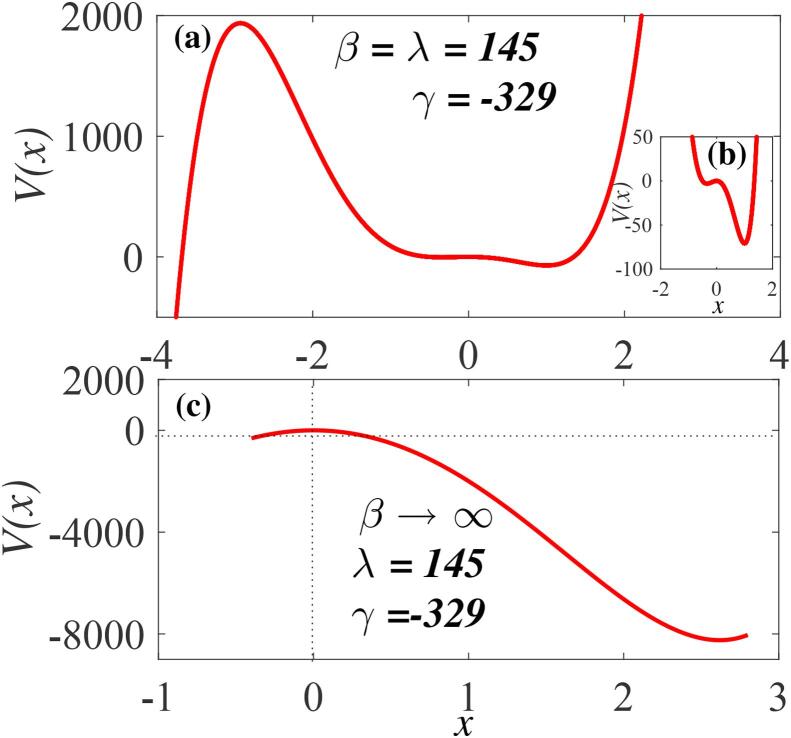
The bubble oscillator potential V(x) from Eq. (14): (a) double-well for β>0,γ<0 and λ>0, (two stable equilibria and two unstable equilibria). The parameters are λ=145 and γ=-329. (b) Zoom of the asymmetric double-well potential in (a). (c) Point of no equilibrium with other parameters fixed as in (a) for β→∞, (point of inflection with β=4000).

The completeness of the proposed prediction is clearly summarised in Fig. 2, Fig. 3 where we have plotted the potential V(x) for different values of β,γ and λ. A single-well (β<0,γ=λ=0) or a single-hump (β>0,γ=λ=0) as shown in Fig. 2(a) illustrates the special case of V(x) in which γ=λ=0, corresponding to a harmonic oscillator,

$$\overset{¨}{x}+\alpha_{d}\overset{˙}{x}+\beta_{0}x=0,$$

$\alpha_{d}$ and $\beta_{0}$ being respectively the damping parameter and square of the linear resonance frequency investigated earlier [102], [103], [104], [112]. It admits only two types of potential structure satisfying the general *case I* – a single-well for β<0, and a single-hump for β>0.

When higher nonlinear terms are considered two equilibrium states appear. The potential V(x) can be a single-well-single-hump as depicted in Fig. 2(b) for β<0,λ>0,γ<0, corresponding to one stable equilibrium located at x=0 and one unstable equilibrium state located at x=-3, thus satisfying *case II*. Furthermore, an asymmetrical double-well-double-hump for β>0,γ<0,λ>0 is shown in Fig. 3(a). Previous reports had shown that the system potential may consist of a smooth background potential plus a rapidly and randomly varying potential that may alter the dynamics of the system when the particles are insufficiently energetic to overcome the net potential barriers. When a dynamical system with energetic particles encounters such potential, the system is capable of annulling the effects on its trajectories [53]. This case of β>0 (double-well-double-hump potential) is therefore consistent with the stable and unstable equilibria depicted as *case II* provided that the potential barrier at the hump ($H_{1}$) located at x<0 is significantly higher than the barrier ($H_{2}$) near x=0. Under this condition, energetic particles can subdue the potential barrier created in the neighbourhood of this unstable equilibrium ($H_{2}$). The inset, Fig. 3(b) depicts the zoom of the asymmetric structure of the potential well, showing two wells located near x=-0.10 and x=0.35 and sandwiched by relatively small barrier at x=0. Fig. 3(c) shows that our model satisfies *case III* for sufficiently low ambient pressure below Blake’s threshold, showing no equilibrium point. In this case, the particles of the bubble system gain sufficient energy to overcome all the potential barriers due to the infinitely increasing bubble radius as β→∞.

The discussions above can be analytically justified based on theoretical verification of the stability properties using the Jacobian matrix. The unperturbed nonlinear Rayleigh bubble oscillator derived from Eq. (10) is written as:

(15) $$\begin{array}{cc} \overset{˙}{x}= & y\\ \overset{˙}{y}= & \beta x-\gamma x^{2}+\gamma x^{3}-\lambda x^{4}\text{.} \end{array}$$

The equilibrium points, $P_{i}(x_{*},y_{*})$ of the autonomous system (15), where $x_{*}$ and $y_{*}$ are the fixed points are obtained by equating the L.H.S to zero and solving the resultant algebraic equation. Thus,

(16) $$\begin{array}{cc} & y_{*}=0\\ & \beta x_{*}-\gamma x_{*}^{2}+\gamma x_{*}^{3}-\lambda x_{*}^{4}=0\text{.} \end{array}$$

This yields the following four equilibrium points:

(17) $$P_{1}(0,0),P_{2}(\beta ,0),P_{3}\left(\frac{\gamma +\sqrt{\Delta }}{2\lambda },0\right),P_{4}\left(\frac{\gamma -\sqrt{\Delta }}{2\lambda },0\right)\text{.}$$

Remarks 3.1 We make the following remarks on the equilibrium points $P_{i}(i=1,2,3,4)$.

(i) $P_{1}(0,0)$ is a trivial equilibrium point.
(ii) $P_{2}(\beta ,0)$ is a unique equilibrium point and always exists whenever β≠0.
(iii) $P_{3}\left(\frac{\gamma +\sqrt{\Delta }}{2\lambda },0\right)$ and $P_{4}\left(\frac{\gamma -\sqrt{\Delta }}{2\lambda },0\right)$ are non-trivial equilibrium points determined by the quantity $\Delta =\gamma^{2}-4\lambda (\gamma -\beta )$.

Depending on the sign of β, two cases exist for the equilibrium points $P_{2},P_{3}$ and $P_{3}$: (A) when β<0 and (B) when β>0. See the Appendix I for details.

Using the Jacobian matrix at the equilibrium point $P_{i}(x_{*},0)$, we deduce for the equilibrium points that:

(i) $P_{1}(0,0)$ is a saddle when β>0 and a centre when β<0.
(ii) $P_{2}(\beta ,0)$ is a saddle when ζ>0 and a centre when ζ<0, where $\zeta =\beta -2\gamma \beta +3\gamma \beta^{2}-4\lambda \beta^{3}$.
(iii) $P_{3}\left(\frac{\gamma +\sqrt{\Delta }}{2\lambda },0\right)$ is a saddle if ϖ>0 and a centre if ϖ<0; where ϖ is defined in the Appendix I.
(iv) $P_{4}\left(\frac{\gamma -\sqrt{\Delta }}{2\lambda },0\right)$ is a saddle if ξ>0 and a centre if ξ<0;ξ is defined in the Appendix I.

It is conjectured that the system can also admit additional equilibrium solutions depending on the choice of truncation of the binomial expansion of ${\left(1+x\right)}^{-1}$ in connection with higher-order nonlinear terms of the potential functions. Research shows that up-to-triple well solutions are possible in higher-order nonlinear systems in contrast with the familiar single and double equilibrium solutions reported earlier for lower-order potential functions [53], [115], [123], [124], [125]. Similarly, a flip-flop between hard-spring and soft-spring bistabilities due to higher-order truncation of the Toda oscillator was observed and analyzed by Goswami [126] while, earlier, a third-order approximation reduced the Toda oscillator model to the Heńon–Heiles (HH) Hamiltonian system which is non-integrable in contrast to the original integrable Toda Hamiltonian [127], [128], [129]. Although the nonlinear dynamics, including the bifurcations and the evolution of diverse orbits as well as details of the equilibrium points of our derived bubble oscillator, is beyond the scope of the present paper, we comment that the features of our derived bubble potential function are consistent with the previously reported results [116], [117], [118], [119], [130], [131], [121].

## 4 Acoustic vibrational resonance

Now, we return to the main focus of this paper. The bubble oscillations can be highly complex and chaotic. We note that the media and the type of application determine parameters such as the density, viscosity, surface tension, and diameter of the bubble [132]. To investigate the occurrence of VR in Eq. (10), a higher amplitude and fast periodic driving force is coupled to the acoustic sound field, such that the low-amplitude acoustic field is modulated by a high-frequency cosine signal, fcosΩt. Under the exposure of the high-frequency modulating signal, a single bubble emit light - a phenomenon known as single bubble sonoluminescence (SBSL). Thus, our choice of high-frequency modulation signal stems from their roles in SBSL and other real-world bubble cavitation experimental applications in which only certain parameters are available for perturbation. Hence in practice we can only expect to perturb the external forcing terms. Parameters such as the surface tension and liquid viscosity are strictly determined by the host medium and therefore not available for perturbation since it would be impracticable e.g. to open up the skin during high-intensity focused ultrasound (HIFU) tumor treatment to change the properties of the tumor medium [132]. Here, we define Ω≫ω, and its amplitude is greater than that of the weak driving acoustic force. In the following analysis, we denote by η the coefficient of the quadratic damping force in Eq. (10) to obtain,

(18) $$\begin{array}{cc} \overset{¨}{x}+ & \overset{˙}{x}[\alpha_{0}-\alpha_{1}x+\alpha_{2}x^{2}-\alpha_{3}x^{3}+\alpha_{4}x^{4}]\\ + & \eta {\overset{˙}{x}}^{2}(1-x+x^{2})-x(\beta -\delta (f\cos\Omega t+∊)\cos\omega t)\\ + & x^{2}(\gamma -\delta (f\cos\Omega t+∊)\cos\omega t)\\ - & \gamma x^{3}+\lambda x^{4}=\delta (f\cos\Omega t+∊)\cos\omega t \end{array}$$

(19) $$\begin{array}{cc} \overset{¨}{x}= & -\overset{˙}{x}[\alpha_{0}-\alpha_{1}x+\alpha_{2}x^{2}-\alpha_{3}x^{3}+\alpha_{4}x^{4}]\\ - & \eta {\overset{˙}{x}}^{2}(1-x+x^{2})+x(\beta -\delta (f\cos\Omega t+∊)\cos\omega t)\\ - & x^{2}(\gamma -\delta (f\cos\Omega t+∊)\cos\omega t)\\ + & \gamma x^{3}-\lambda x^{4}+\delta (f\cos\Omega t+∊)\cos\omega t\text{.} \end{array}$$

### 4.1 Analytical description of VR

We now employ the method of direct separation of the dynamics, to separate the motion of the bubble system into fast and slow motions. Thus, we obtain a set of integro-differential equations consisting of the systems’ equation of slow motion whose response can be varied by simply adjusting the parameters of the high-frequency driving input. The response amplitude, *Q*, defined as the ratio of the amplitude $A_{L}$ to the frequency *f* is obtained by solving the equation of slow motion. Thus the solution x(t) of the bubble oscillator given by (18) is assumed to be a superposition of the solutions χ(t) for the slow dynamics whose frequency is ω and of ψ(t) for the fast motion with frequency Ω with Ω≫ω, in the form:

(20) x(t)=χ(t)+ψ(t,Ωt).

Here χ(t) is a periodic function of period $T=\frac{2\pi }{\omega }$ and ψ is a periodic function in the fast time τ=Ωt with period 2π. The mean value of ψ with respect to fast time τ is given by

(21) $$\langle \psi \rangle =\frac{1}{2\pi }\int_{0}^{2\pi }\psi \;d\tau =0\text{.}$$

The next step is to derive a system of two coupled integro-differential equations for the variables χ and ψ from the main equation of the system (18), though our main interest will be in the slow component. By substituting Eq. (20) into Eq. (18), we obtained the first of the two equations:

(22) $$\begin{array}{cc} \overset{¨}{\chi }+ & \overset{¨}{\psi }+(\overset{˙}{\chi }+\overset{˙}{\psi })[\alpha_{0}-\alpha_{1}(\chi +\psi )+\alpha_{2}{\left(\chi +\psi \right)}^{2}\\ - & \alpha_{3}{\left(\chi +\psi \right)}^{3}+\alpha_{4}{\left(\chi +\psi \right)}^{4}]+\eta {\left(\overset{˙}{\chi }+\overset{˙}{\psi }\right)}^{2}(1-(\chi +\psi )\\ + & {\left(\chi +\psi \right)}^{2})-(\chi +\psi )(\beta -\delta (f\cos\Omega t+∊)\cos\omega t)\\ + & {\left(\chi +\psi \right)}^{2}(\gamma -\delta (f\cos\Omega t+∊)\cos\omega t)-\gamma {\left(\chi +\psi \right)}^{3}\\ + & \lambda {\left(\chi +\psi \right)}^{4}=\delta (f\cos\Omega t+∊)\cos\omega t\text{.} \end{array}$$

Expanding Eq. (22), we have

(23) $$\begin{array}{cc} \overset{¨}{\chi }+ & \overset{¨}{\psi }+\overset{˙}{\chi }[\alpha_{0}-\chi (\alpha_{1}-2\alpha_{2}\psi +3\alpha_{2}\psi^{3}-4\alpha_{4}\psi^{3})\\ + & \chi^{2}(\alpha_{2}-3\alpha_{3}\psi +6\alpha_{4}\psi^{2})-\chi^{3}(\alpha_{3}-4\alpha_{4}\psi )\\ + & \alpha_{4}\chi^{4}-\alpha_{1}\psi +\alpha_{2}\psi^{2}-\alpha_{3}\psi^{3}+\alpha_{4}\psi^{4}]+\overset{˙}{\psi }[\alpha_{0}\\ - & \chi (\alpha_{1}-2\alpha_{2}\psi +3\alpha_{3}\psi^{3}-4\alpha_{4}\psi^{3})\\ + & \chi^{2}(\alpha_{2}-3\alpha_{3}\psi +6\alpha_{4}\psi^{2})-\chi^{3}(\alpha_{3}-4\alpha_{4}\psi )\\ + & \alpha_{4}\chi^{4}-\alpha_{1}\psi +\alpha_{2}\psi^{2}-\alpha_{3}\psi^{3}+\alpha_{4}\psi^{4}]\\ + & \eta {\overset{˙}{\chi }}^{2}[1-\chi (1-2\psi )+\chi^{2}-\psi +\psi^{2}]\\ + & \eta {\overset{˙}{\psi }}^{2}[1-\chi (1-2\psi )+\chi^{2}-\psi +\psi^{2}]\\ + & 2\eta \chi \psi [1-\chi (1-2\psi )+\chi^{2}-\psi +\psi^{2}]\\ - & \chi [\beta -\delta (f\cos\Omega t+∊)\cos\omega t]\\ - & \psi [\beta -\delta (f\cos\Omega t+∊)\cos\omega t]\\ + & \chi^{2}[\gamma -\delta (f\cos\Omega t+∊)\cos\omega t]\\ + & \psi^{2}[\gamma -\delta (f\cos\Omega t+∊)\cos\omega t]\\ + & 2\chi \psi [\gamma -\delta (f\cos\Omega t+∊)\cos\omega t]\\ - & \gamma [\chi^{3}+3\chi^{2}\psi +3\chi \psi^{2}+\psi^{3}]\\ + & \lambda [\chi^{4}+4\chi^{3}\psi +6\chi^{2}\psi^{2}+4\chi \psi^{3}+\psi^{4}]\\ = & \delta (f\cos\Omega t+∊)\cos\omega t\text{.} \end{array}$$

By averaging Eq. (23), and using the following definitions of averages

(24) $$\begin{array}{cc} & \langle \psi \rangle =\overset{¯}{\psi }=\frac{1}{2\pi }\int_{0}^{2\pi }\psi d\tau =0\\ & \overset{¯}{\overset{¨}{\psi }}=\overset{¯}{\overset{˙}{\psi }}=0\\ & \langle f\cos\Omega t\rangle =\overset{¯}{f\cos\Omega t}=0, \end{array}$$

Eq. (23) reduces to

(25) $$\begin{array}{cc} \overset{¨}{\chi }+ & \overset{˙}{\chi }[\alpha_{0}-\chi (\alpha_{1}+3\alpha_{3}\overset{‾}{\psi^{2}}-4\alpha_{4}\overset{‾}{\psi^{3}})+\chi^{2}(\alpha_{2}+6\alpha_{4}\overset{‾}{\psi^{2}})\\ - & \alpha_{3}\chi^{3}+\alpha_{4}\chi^{4}+\alpha_{2}\overset{‾}{\psi^{2}}-\alpha_{3}\overset{‾}{\psi^{3}}+\alpha_{4}\overset{‾}{\psi^{4}}]\\ + & \eta {\overset{˙}{\chi }}^{2}[1-\chi +\chi^{2}+\overset{‾}{\psi^{2}}]+\eta \overset{‾}{{\overset{˙}{\psi }}^{2}}[1-\chi +\chi^{2}+\overset{‾}{\psi^{2}}]\\ + & \chi^{2}[\gamma -\delta ∊\cos\omega t]+\overset{‾}{\psi^{2}}[\gamma -\delta ∊\cos\omega t]\\ - & \gamma [\chi^{3}+3\chi \overset{‾}{\psi^{2}}+\overset{‾}{\psi^{3}}]-\chi [\beta -\delta ∊\cos\omega t]\\ + & \lambda [\chi^{4}+6\chi^{2}\overset{‾}{\psi^{2}}+4\chi \overset{‾}{\psi^{3}}+\overset{‾}{\psi^{4}}]=\delta ∊\cos\omega t\text{.} \end{array}$$

Eq. (25) is the sought-after analytic expression for the slow oscillation of the bubble, with the parameters of the fast signal. Eq. (25) will be employed in computing the theoretical response amplitude *Q* of the bubble system at the lower frequency ω. To obtain the equation of fast motion, we subtract Eq. (25) from Eq. (23) to get

(26) $$\begin{array}{cc} \overset{¨}{\psi }+ & \overset{˙}{\psi }[\alpha_{0}-\chi (\alpha_{1}-2\alpha_{2}\psi +3\alpha_{3}\psi^{3}-4\alpha_{4}\psi^{3})\\ + & \chi^{2}(\alpha_{2}-3\alpha_{3}\psi +6\alpha_{4}\psi^{2})-\chi^{3}(\alpha_{3}-4\alpha_{4}\psi )+\alpha_{4}\chi^{4}\\ - & \alpha_{1}\psi +\alpha_{2}\psi^{2}-\alpha_{3}\psi^{3}+\alpha_{4}\psi^{4}]+\overset{˙}{\chi }[-\chi (-2\alpha_{2}\psi \\ + & 3\alpha_{3}(\psi^{2}-\overset{‾}{\psi^{2}})-4\alpha_{4}(\psi^{3}-\overset{‾}{\psi^{3}}))+\chi^{2}(-3\alpha_{3}\psi \\ + & 6\alpha_{4}(\psi^{2}-\overset{‾}{\psi^{2}}))+4\alpha_{4}\chi^{3}\psi -\alpha_{1}\psi +\alpha_{2}(\psi^{2}-\overset{‾}{\psi^{2}})\\ - & \alpha_{3}(\psi^{3}-\overset{‾}{\psi^{3}})+\alpha_{4}(\psi^{4}-\overset{‾}{\psi^{4}})]+\eta {\overset{˙}{\chi }}^{2}[\chi \psi -\psi +(\chi^{2}-\overset{‾}{\psi^{2}})]\\ + & \eta ({\overset{˙}{\psi }}^{2}-\overset{‾}{{\overset{˙}{\psi }}^{2}})[-2\alpha_{2}\psi -\psi +(\psi^{2}-\overset{‾}{\psi^{2}})]2\eta \chi \psi [1\\ - & \chi (1-2\psi )\chi^{2}-\psi +\psi^{2}]+\chi [\delta (f\cos\Omega t)\cos\omega t]\\ - & \psi [\beta -\delta (f\cos\Omega t+∊)\cos\omega t]+\chi^{2}[\delta (f\cos\Omega t)\cos\omega t]\\ + & (\psi^{2}-\overset{‾}{\psi^{2}})[\gamma -\delta (f\cos\Omega t)\cos\omega t]+2\chi \psi [\gamma \\ - & \delta (f\cos\Omega t+∊)\cos\omega t]-\gamma [3\chi^{2}\psi +3\chi (\psi^{2}-\overset{‾}{\psi^{2}})\\ + & \left(\psi^{3}-\overset{‾}{\psi^{3}})]+\lambda [4\chi^{3}\psi +6\chi^{2}(\psi^{2}-\overset{‾}{\psi^{2}}\right)\\ + & 4\chi (\psi^{3}-\overset{‾}{\psi^{3}})+(\psi^{4}-\overset{‾}{\psi^{4}})]=\delta (f\cos\Omega t)\cos\omega t, \end{array}$$

where

(27) $$\begin{array}{cc} \psi = & \frac{-f}{\Omega^{2}}\cos\Omega t,\;\;\overset{‾}{{\overset{˙}{\psi }}^{2}}=\frac{f^{2}}{2\Omega^{2}},\\ \overset{‾}{\psi^{2}}= & \frac{f^{2}}{2\Omega^{4}},\;\overset{‾}{\psi^{4}}=\frac{3f^{4}}{8\Omega^{8}},\;\overset{‾}{\psi }=\overset{‾}{\psi^{3}}=\overset{‾}{\psi^{5}}\ldots \;=0\text{.} \end{array}$$

Substituting Eq. (27) into Eq. (25), we can then express Eq. (26) as:

(28) $$\begin{array}{cc} \overset{¨}{\chi }+ & \overset{˙}{\chi }[C_{0}-C_{1}\chi +C_{2}\chi^{2}-\alpha_{3}\chi^{3}+\alpha_{4}\chi^{4}]\\ + & \eta {\overset{˙}{\chi }}^{2}[C_{3}-\chi +\chi^{2}]-\chi [C_{4}-\delta ∊\cos\omega t]\\ + & \chi^{2}[C_{5}-\delta ∊\cos\omega t]-\gamma \chi^{3}\\ + & \lambda \chi^{4}+[C_{6}-C_{7}∊\cos\omega t]=\delta ∊\cos\omega t, \end{array}$$

where

(29) $$\begin{array}{cc} C_{0}= & (\alpha_{0}+\frac{\alpha_{2}f^{2}}{2\Omega^{4}}+\frac{3\alpha_{4}f^{2}}{8\Omega^{4}}),C_{1}=(\alpha_{1}+\frac{3\alpha_{3}f^{2}}{2\Omega^{4}}),\\ C_{2}= & (\alpha_{2}+\frac{3\alpha_{4}f^{2}}{\Omega^{4}}),C_{3}=(1+\frac{f^{2}}{2\Omega^{4}}),\\ C_{4}= & (\frac{\eta f^{2}}{2\Omega^{2}}+\beta +\frac{3\gamma f^{2}}{2\Omega^{4}}),C_{5}=(\frac{\eta f^{2}}{2\Omega^{2}}+\gamma +\frac{3\lambda f^{2}}{\Omega^{4}}),\\ C_{6}= & (\frac{\eta f^{2}}{2\Omega^{2}}+\frac{\eta f^{4}}{4\Omega^{10}}+\frac{\gamma f^{2}}{2\Omega^{4}}+\frac{3\lambda f^{4}}{2\Omega^{8}}),\mathrm{and}C_{7}=(\frac{\delta f^{2}}{2\Omega^{4}})\text{.} \end{array}$$

Thus, we can write Eq. (28) as

(30) $$\begin{array}{cc} \overset{¨}{\chi }+\overset{˙}{\chi }[C_{0}- & C_{1}\chi +C_{2}\chi^{2}-\alpha_{3}\chi^{3}+\alpha_{4}\chi^{4}]\\ + & \eta {\overset{˙}{\chi }}^{2}[C_{3}-\chi +\chi^{2}]-\frac{\mathrm{dV}(\chi )}{d\chi }=\delta ∊\cos\omega t, \end{array}$$

where $V_{\mathrm{eff}}(\chi )$, the effective potential of the system, is given as

(31) $$\begin{array}{c} V_{\mathrm{eff}}(\chi )=[C_{6}-C_{7}∊\cos\omega t]\chi +\frac{\left[C_{4}-\delta ∊\cos\omega t\right]}{2}\chi^{2}\;\\ -\frac{\left[C_{5}-\delta ∊\cos\omega t\right]}{3}\chi^{3}+\frac{\gamma }{4}\chi^{4}-\frac{\lambda }{5}\chi^{5}\;\;\; \end{array}$$

and $C_{4}$ is the effective natural frequency defined in Eq. (29). The deviation of the slow motion χ from $\chi^{*}$ is obtained by substituting $Y=\chi -\chi^{*}$ in Eq. (28) so that

(32) $$\begin{array}{cc} \overset{¨}{Y}+ & C_{0}\overset{˙}{Y}+\overset{˙}{Y}[-C_{1}(Y+\chi^{*})+C_{2}{\left(Y+\chi^{*}\right)}^{2}\\ - & \alpha_{3}{\left(Y+\chi^{*}\right)}^{3}+\alpha_{4}{\left(Y+\chi^{*}\right)}^{4}]\\ + & \eta {\overset{˙}{Y}}^{2}[C_{3}-(Y+\chi^{*})+{\left(Y+\chi^{*}\right)}^{2}]\\ - & Y[C_{4}-\delta ∊\cos\omega t]+Y^{2}[C_{5}-\delta ∊\cos\omega t]\\ + & 2Y\chi^{*}[C_{5}-\delta ∊\cos\omega t]-\gamma Y^{3}+\lambda Y^{4}\\ - & \chi^{*}[C_{4}-\delta ∊\cos\omega t]+\chi^{*2}[C_{5}-\delta ∊\cos\omega t]\\ - & 3\gamma Y^{2}\chi^{*}-3\gamma Y\chi^{*2}-\gamma \chi^{*3}+4\lambda Y^{3}\chi^{*}\\ + & 6\lambda Y^{2}\chi^{*2}+4\lambda Y\chi^{*3}+\lambda \chi^{*4}\\ + & [C_{6}-C_{7}∊\cos\omega t]=\delta ∊\cos\omega t\text{.} \end{array}$$

For ∊≪1, we can assume that |Y|≪1. Thus, if the oscillation takes place around the equilibrium $\chi^{*}=0$, then Eq. (32) becomes

(34) $$\begin{array}{cc} \overset{¨}{Y}+ & C_{0}\overset{˙}{Y}+\overset{˙}{Y}[-C_{1}Y+C_{2}Y^{2}-\alpha_{3}Y^{3}+\alpha_{4}Y^{4}]\\ + & \eta {\overset{˙}{Y}}^{2}[C_{3}-Y+Y^{2}]-Y[C_{4}-\delta ∊\cos\omega t]\\ + & Y^{2}[C_{5}-\delta ∊\cos\omega t]-\gamma Y^{3}+\lambda Y^{4}\\ + & [C_{6}-C_{7}∊\cos\omega t]=\delta ∊\cos\omega t\text{.} \end{array}$$

Hence, by neglecting the nonlinear term in Eq. (34), we obtain a linear system written as

(35) $$\overset{¨}{Y}+C_{0}\overset{˙}{Y}-Y[C_{4}-\delta ∊\cos\omega t]=(\delta +C_{7})∊\cos\omega t-C_{6}\;\;$$

which can be re-written as

(36) $$\overset{¨}{Y}+C_{0}\overset{˙}{Y}-\omega_{r}^{2}Y=F\cos\omega t,$$

where, $\omega_{r}={\left(C_{4}-\delta ∊\cos\omega t\right)}^{\frac{1}{2}}$ and $F=∊(\delta +C_{7})$ .

Eq. (36) has a steady-state solution $Y(t)=A_{L}\cos(\omega t+\phi )$ in the limit *t* approaches ∞; and the response amplitude $A_{L}$ is then given as

(37) $$A_{L}=\frac{F}{\sqrt{{\left(\omega_{r}^{2}-\omega^{2}\right)}^{2}+C_{0}^{2}\omega^{2}}},\phi =-\arctan\frac{C_{0}\omega }{\left(\omega^{2}-\omega_{r}^{2}\right)}$$

such that,

(38) $$Q=\frac{A_{L}}{F}=\frac{1}{\sqrt{{\left(\omega_{r}^{2}-\omega^{2}\right)}^{2}+C_{0}^{2}\omega^{2}}}\text{.}$$

**Fig. 4:**
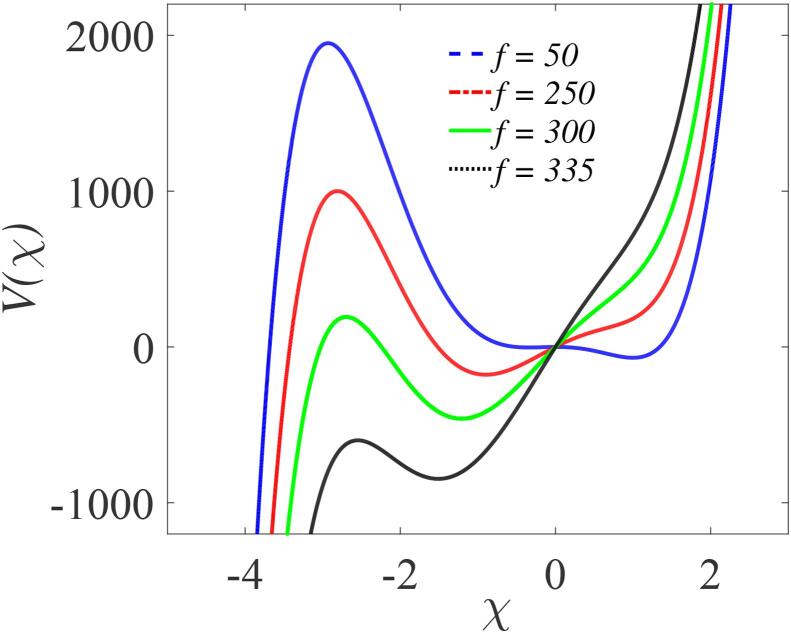
The effective potential of the system computed from Eq. (31), with four values of f(=50,250,300,335and300) and other parameters are β=λ=145,δ=-10,Ω=10ω,ω=5,∊=1, and γ=-329 with $t=10^{6}$.

Fig. 4 plots the effective potential $V_{\mathrm{eff}}(\chi )$ as a function of χ, the component of the slow motion, for different amplitudes (f=50,200,300, and 335 ) of the fast periodic force, with parameters β=λ=145,δ=-10,Ω=10ω,ω=5, and γ=-392 fixed. As shown in Fig. 4, $V_{\mathrm{eff}}(\chi )$ is a double-well for the choice of parameter values considered, similar to the potential shown in Fig. 3(a) for zero and small amplitudes, *f*. With increasing *f* the shape of the potential changes and, for f=335, it becomes a single well with no resemblance to the system’s potential presented in Fig. 3(a). It is evident that the effective potential of the slow motion depends on the amplitude *f* and frequency Ω of the fast motion. In the absence of a high frequency driving force, i.e. by setting the amplitude f=0 in Eqs. (26), then Eq. (31) reduces to Eq. (13), the model’s potential.

One of the most striking results of this paper is that there is also a contribution to the effective resonance frequency of the system due to the parameters of the fast-oscillating force as given by Eq. (28). The effective resonance frequency is therefore $\left(\frac{\eta f^{2}}{2\Omega^{2}}+\beta +\frac{3\gamma f^{2}}{2\Omega^{4}}\right)$. This contribution was either not found, or more usually overlooked, in most earlier analyses of VR. Thus, our results here differ significantly from previous ones, where VR was found in systems with linear and non-linear dissipation, such as the asymmetric Duffing and plasma oscillators, in which VR has been extensively studied. For small amplitude oscillations, the dynamics of the oscillating gas bubble explicitly depends on the acoustic damping constant and the natural resonance frequency of the bubble motion [44], [111], [133], [134]. We remark at this point, that our interest in the parameter of the natural resonance frequency of the system is that it principally defines the various types of bubble resonance (e.g. main resonance, harmonics, subharmonics and combination resonances) observed under multi-frequency acoustic excitation [44]. In addition, it dictates the shape of the potential wells of the system.

We will consider both the single-well and double-well configurations of the system’s potential, noting that in the latter case there is the possibility of observing multiple resonance peaks, and bearing in mind that VR occurs in systems with bistable and multistable potentials where for certain parameter regimes multiple resonance can be obtained [1], [13], [14], [18], [55], [61], [62], [135].

Note that the effective resonance frequency can play a direct and significant role analogous to the effective nonlinear dissipation reported in Refs [7], [10], [54], [53] in the enhancement of signals by modulating the parameters of the fast signal. The effective resonance frequency parameters, which can be computed from the second term of Eq. (31), therefore dictate the equilibrium points of the slow motion. When ∊=0 in Eq. (31), the effective potential function reduces to,

(39) $$V_{\mathrm{eff}}(\chi )=-C_{6}\chi +C_{4}\frac{\chi^{2}}{2}-\frac{C_{5}\chi^{3}}{3}+\frac{\gamma \chi^{4}}{4}+\frac{\lambda \chi^{5}}{5}\text{.}$$

The shape, the number of local extrema and their locations in the system’s potential V(x), given by Eq. (14), depend on the parameters δ,β,γ and λ. For the effective potential $V_{\mathrm{eff}}(\chi )$, however, these properties depend also on the parameters *f* and Ω. In Eq. (14), there are two stable equilibrium points plus x=0 as a third equilibrium point, while in the effective potential $V_{\mathrm{eff}}(\chi )$ given by Eq. (39), the number of equilibrium points increases to four besides the trivial equilibrium point, $\chi^{*}$ (See Appendix II). Consequently, by varying either *f* or Ω, new equilibrium states can be created and the number of equilibrium states can be changed.

### 4.2 Numerical description of VR

For full numerical integration of the nonlinear Rayleigh bubble oscillator, it is expedient to write Eq. (19) as a set of two coupled, first-order, autonomous, ordinary differential equations (ODEs) of the form;

(40) $$\begin{array}{cc} \frac{\mathrm{dx}}{\mathrm{dt}}= & y\\ \frac{\mathrm{dy}}{\mathrm{dt}}= & -y[\alpha_{0}-\alpha_{1}x+\alpha_{2}x^{2}-\alpha_{3}x^{3}+\alpha_{4}x^{4}]\\ - & \eta y^{2}(1-x+x^{2})+x(\beta -\delta (f\cos\Omega t+∊)\cos\omega t)\\ - & x^{2}(\gamma -\delta (f\cos\Omega t+∊)\cos\omega t)\\ + & \gamma x^{3}-\lambda x^{4}+\delta (f\cos\Omega t+∊)\cos\omega t, \end{array}$$

where the response *Q* of the system can be computed from the expression

(41) $$Q=\frac{\sqrt{A_{S}^{2}+A_{C}^{2}}}{f},$$

(42) $$\phi =-\tan^{-1}\left(\frac{A_{S}}{A_{C}}\right),$$

such that the response to the frequency ω is defined as the amplitude of the sine and cosine components of the output signal, and the numerical values of $A_{S}$ and $A_{C}$ are related to the Fourier spectrum of the time series of the variable *x* computed at the frequency ω as

(43) $$\begin{array}{c} A_{S}=\frac{2}{\mathrm{nT}}\int_{0}^{\mathrm{nT}}x(t)\sin\omega t\;\mathrm{dt}=0,\\ A_{C}=\frac{2}{\mathrm{nT}}\int_{0}^{\mathrm{nT}}x(t)\cos\omega t\;\mathrm{dt}=0\text{.} \end{array}$$

In Eq. (43), $T=\frac{2\pi }{\omega }$ is the oscillation period of the low frequency input signal with n=(1,2,3…) being the number of complete oscillations. Zero initial conditions were used in computations throughout the paper, and the parameters $\alpha_{0}=0.08,\alpha_{1}=0.12,\alpha_{2}=0.16,\alpha_{3}=0.08$ and $\alpha_{4}=0.04$ were kept fixed.

## 5 Analyses of resonance

We now analyze vibrational resonance in the bubble oscillator under different sets of conditions. The amplitude of the system’s response is $Q=\frac{1}{\sqrt{S}}$; where $S={\left(\omega_{r}^{2}-\omega^{2}\right)}^{2}+C_{0}^{2}\omega^{2}$from Eq. (38). In the linearized equation of the system, the linear dissipation $C_{0}$ appearing in Eq. (36) is the linear damping coefficient which, for a given liquid of density ρ, is dependent on both the frequency Ω, and the amplitude *f*, of the fast signal, as well as on the fundamental parameters of the medium (i.e. the thermal ($\mu_{\mathrm{th}})$ and liquid ($\mu_{l}$) viscosities, respectively). Moreover, the contribution of the damping force is negligible in determining the dynamics of the system compared to the effect of the liquid’s surface tension, the related natural frequency of the system (β); and variations in the parameters of the fast signal (*g* or Ω) produce the same effect as β. It follows that a variation in the parameters on which the natural frequency of the oscillating gas bubble depends would produce additive and multiplicative effects on the appearance of VR in the system in accordance with $C_{0}$.

We set $W=\omega_{r}^{2}-\omega^{2}$, so that $S=W^{2}+C_{0}\omega^{2}$ suggests that *Q* will attain its maximum when *S* is minimum, that is, when W=0 (such that, $\omega_{r}=\omega$, where $\omega_{r}=\sqrt{\left(C_{4}-\delta ∊\cos\omega t\right)}$, with $C_{4}=(\frac{\eta f^{2}}{2\Omega^{2}}+\beta +\frac{3\gamma f^{2}}{2\Omega^{4}})$), and ω≪Ω. Consequently, resonance will occur. For ω→∞,the value of Ω becomes too large (i.e. since Ω≫ω) so that $C_{0}\to 0$, and the system reduces to a linear differential equation with no dissipation, implying that VR does not occur.

Since the acoustic wave is weak (∊≪1), and the amplitude of the bubble oscillation is considered to be small [111], $\omega_{r}=\sqrt{C_{4}}$ because the choice of ω is always very small compared to Ω and δ<0. Consequently, $\omega_{r}^{2}=\frac{\eta f^{2}}{2\Omega^{2}}+\beta +\frac{3\gamma f^{2}}{2\Omega^{4}}$, so that $\omega_{r}=\pm \sqrt{\beta +\frac{f^{2}}{2\Omega^{2}}(\eta +\frac{3\gamma }{\Omega^{2}})}$, allowing us to identify two distinct resonance cases for our system.

Case 1 Single-well condition, for β<0,γ<0 and λ>0, when
  (44) $$\beta ⩽\frac{f^{2}}{2\Omega^{2}}\left(\eta +\frac{3\gamma }{\Omega^{2}}\right)\text{.}$$
Case 2 Double-well condition, for β>0,γ<0 and λ>0, when
  (45) $$\beta ⩾\frac{f^{2}}{2\Omega^{2}}\left(\eta +\frac{3\gamma }{\Omega^{2}}\right)\text{.}$$

Eqs. (31), (39) enable us to analyse the conditions for the occurrence of VR in each case. This shows that single, double, or multiple VR peaks can occur in the system under both conditions. Moreover, the appearance of the resonance peaks could be achieved by adjusting either the parameters of the fast signal (*f* or Ω), or the parameters on which the natural frequency of the bubble oscillator depends β.

### 5.1 Resonance with a single-well potential

We fix parameter values as follows: $\alpha_{0}=0.08,\alpha_{1}=0.12,\alpha_{2}=0.16,\alpha_{3}=0.08,\alpha_{4}=0.04$ and η=1.5. We choose β<0,γ<0 and λ>0, for which V(x) is a single-well potential as shown in Fig. 3(a). In order to examine the response amplitude of the system, Eqs. (41) was computed from the numerically integrated Eq. (43) using the solutions of the Eq. (19) obtained earlier. To validate the analysis, the theoretical *Q* was obtained by integrating Eq. (34) and the results compared with *Q* obtained directly from Eq. (41).

**Fig. 5:**
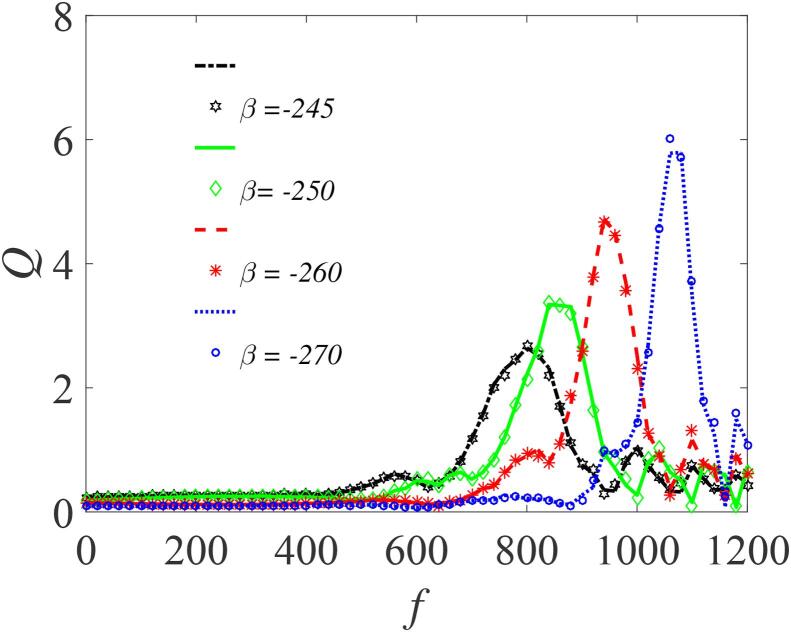
Dependence of *Q* on *f* for single well conditions, with other parameters fixed at Ω=20ω,ω=15,∊=0.01,λ=145,δ=-2 and γ=-32.9. Continuous curves or broken lines represent the numerically-computed *Q* from Eq. (19) using Eq. (42), while the analytically calculated *Q* from Eqs. (34) and (42), are indicated by marker points.

**Fig. 6:**
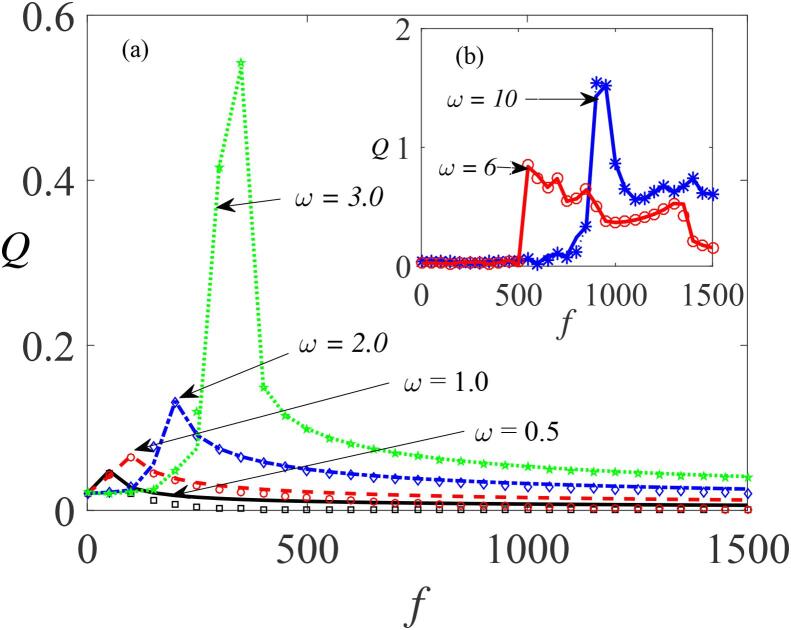
Response amplitude, *Q* against *f* for different values of ω=0.5,1.0,2.0,and 3.0 with Ω=20ω,β=-245,∊=0.01,λ=145,δ=-2 and γ=-32.9. Continuous curves or broken lines represent the numerically-computed *Q* from Eq. (19) using Eq. (42), while the analytically calculated *Q* from Eqs. (34) and (42), are indicated by marker points.

Fig. 5 shows *Q* plotted against the amplitude *f* of the fast periodic force, for four values of β (-245,-250,-260,and -270) obtained from Eq. (34), and superimposed with their corresponding numerical curves computed from Eq. (19) for comparison. It is clear that the theoretical and numerical results are in good agreement. Here, we treat β as the control parameter for a fixed value of the quantities Ω=20ω,ω=15,∊=0.01,λ=145,δ=-2 and γ=-32.9. There is a marked increase in response amplitude as the value of βdecreases. In Fig. 5, the resonance peaks appear when f>500. This is because the resonance frequency, $\omega_{r}\to 0$ when the value of ω is appreciably large since Ω≫ω and β<0, showing that the enhancement of the systems response can also be achieved by modifying either the surface tension of the liquid σ or its density ρ, both of which are dependent on β, as shown by Eq. (10). To further illustrate the occurrence of VR for the conditions of single-well potential, the response *Q* is plotted against *f* in Fig. 6 for different values of ω=0.5,1.0,2.0, and 3.0 with Ω=20ω,β=-245,∊=0.01,λ=145,δ=-2 and γ=-32.9. In this case (Fig. 6(a)), the system also responds significantly to the variation of ω, even at the lower values, e.g. ω=0.5. Here, the resonance peaks occur at lower values of *f* but with smaller magnitudes compared to Fig. 5. Noticeable also is that increase in the values of the frequency of the acoustic signal shifts the peaks rightwards to higher values of *f*, rather than creating more peaks as depicted in Fig. 6(b).

### 5.2 Resonance with double-well potential

**Fig. 7:**
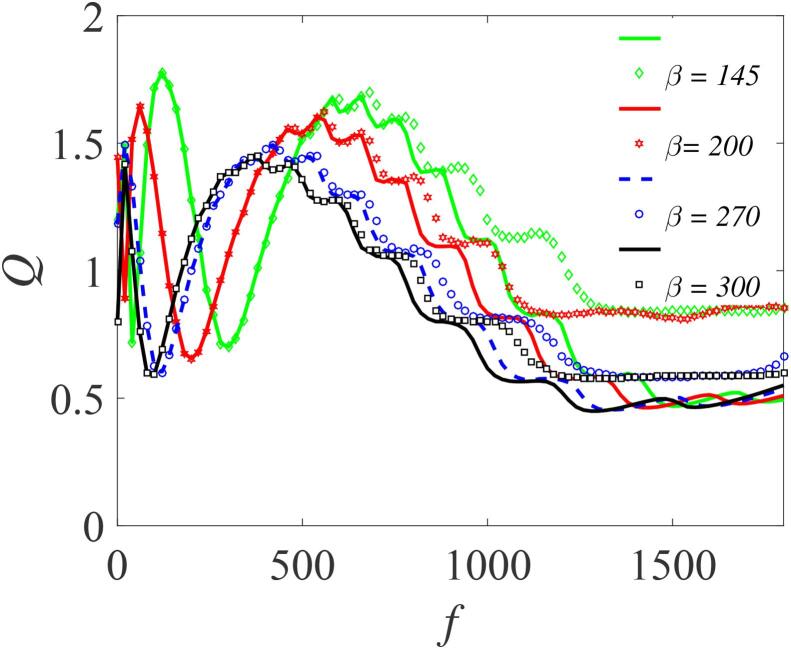
Dependence of *Q* on *f* with the other parameters fixed at Ω=20ω,ω=15,∊=0.01,λ=145,δ=-2 and γ=-32.9. Continuous curves or broken lines represent the numerically-computed *Q* from Eq. (19) using Eq. (42), while the analytically calculated *Q* from Eqs. (34) and (42), are indicated by marker points.

**Fig. 8:**
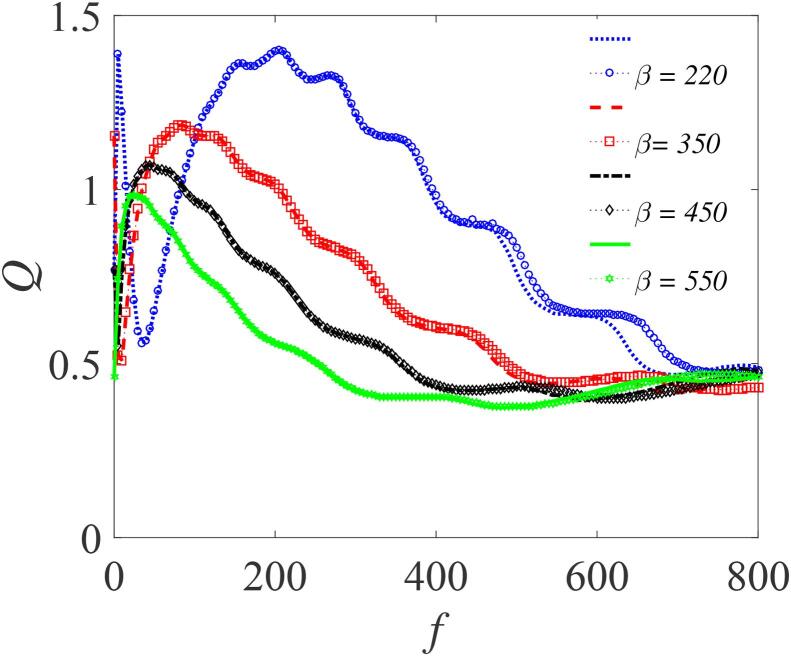
Dependence of *Q* on *f* with varying β=220,350,450 and 550; for Ω=19ω,η=1.5,ω=12,∊=0.01,λ=145,δ=-2 and γ=-32.9. Continuous or broken line represent the numerically-computed *Q* from Eq. (19) using Eq. (42), while the analytically calculated *Q* from Eqs. (34) and (42), are indicated by marker points.

**Fig. 9:**
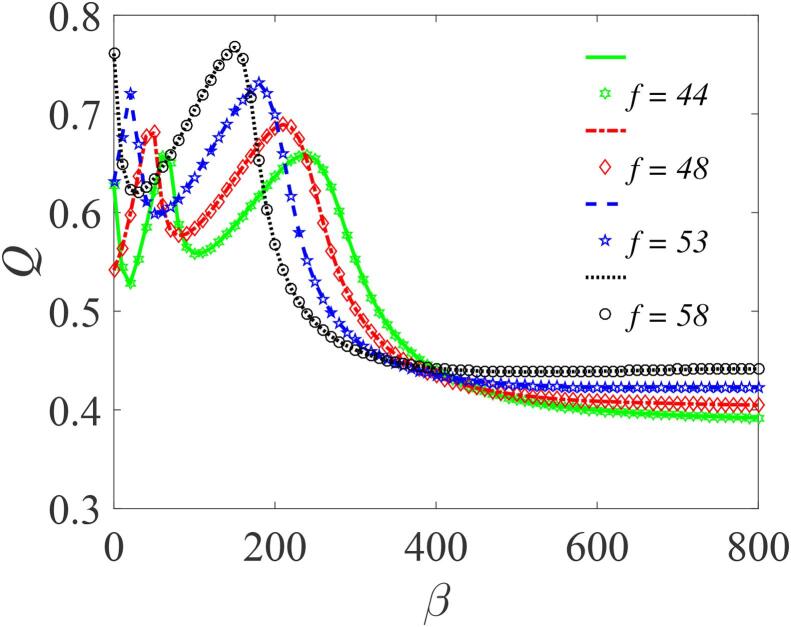
Dependence of *Q* on β with the other parameters fixed at η=1.5,ω=6,∊=0.01,Ω=12ω,λ=145,δ=-7 and γ=-32.9. The theoretical and numerically calculated values of *Q* are represented by marker points and continuous or broken lines respectively.

For the double-well case, subject to the condition: β>0,γ<0 and λ>0, Fig. 7 shows the variation of *Q* as a function of *f* for different values of β, (145,200,270 and 300) with other parameters fixed. Here, *Q* exhibits a number of resonance peaks, with two distinct peaks which approaches the limiting value of 0.4. The response amplitudes *Q* in this case are smaller in magnitude than those in Fig. 5. However, the resonance occurs at lower values of *f*, including the second peaks. Notably, an increase in the value of β produces three remarkable effects: (i) a shift in the resonance peaks towards lower values of *f*; (ii) a reduction in the system’s response; and (iii) elimination of one of the resonance peaks. This is further illustrated in Fig. 8 where we have plotted *Q* against *f* for other values of β and for a different set of the bubble’s parameters. As β becomes larger, typically when β≈550, the small resonance peaks that manifest themselves within the main resonance tend to disappear – a feature that is fully captured in Fig. 9 where *Q* is plotted against β in the weak *f* regimes. For all *f* values shown, two distinct peaks are clearly visible when β<400. However, for β>400, the bubbles cease to vibrate. This can again be inferred from the expression for $\omega_{r}$ at resonance. At resonance, $\omega_{r}=\omega$, and since β>0 such that $\omega_{r}=\sqrt{\frac{\eta f^{2}}{2\Omega^{2}}+\beta +\frac{3\gamma f^{2}}{2\Omega^{4}}}$, it follows that when β→∞, then, $\omega_{r}\gg \omega$ since γ<0. Consequentially, the resonance peaks vanish.

**Fig. 10:**
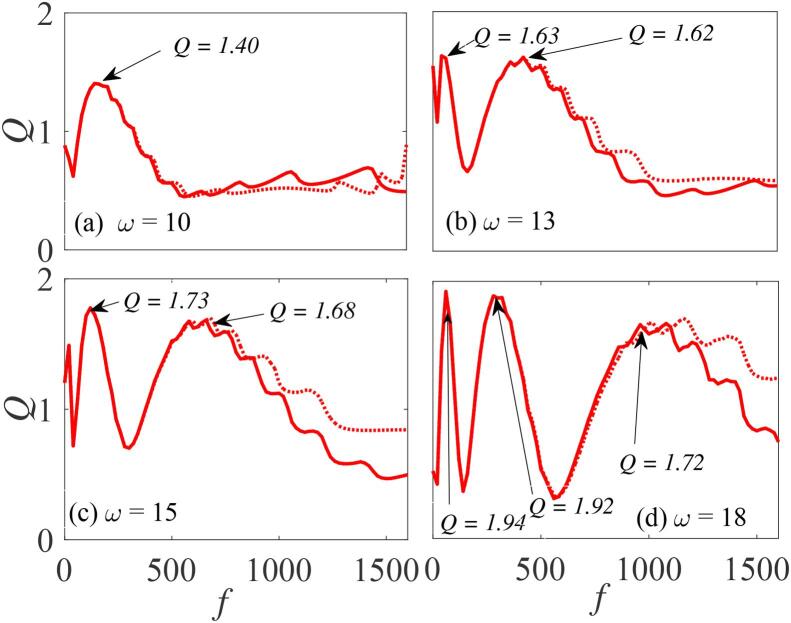
Dependence of *Q* on *f* for (a) ω=10 (b) ω=13 (c) ω=15 and (d) ω=18 with Ω=20ω,β=λ=145,∊=0.01,η=1.5, and γ=-32.9. Continuous curves represent the numerically-computed *Q* from Eq. (19) using Eq. (42), while the analytically calculated *Q* from Eqs. (34) and (42), are indicated by marker points and(or) broken lines.

Fig. 10, shows the dependence of *Q* on *f* for four different values of ω with the following parameters fixed: Ω=20ω,∊=0.01,η=1.5,γ=-32.9, and β=λ=145. It is clear that increasing the value of ω: (i) progressively enhances the system’s response, (ii) shifts the peak positions towards higher values of *f*, and (iii) induces new resonance peaks. For ω=10, only one pronounced resonance peak occurs as shown in Fig. 10(a). In the double-resonance cases shown in Fig. 10(b and c) the two resonance peaks have nearly equal maximum values ($Q_{b1,2}=1.63,1.62$ and $Q_{c1,2}=1.73,1.68$). In Fig. 10(d), when ω=18, three resonance peaks appears with increased response. It has earlier been reported that in pure mechanical systems vibrational resonance occurs in the overdamped case due to minimization of the resonant frequency $\omega_{r}$, while in the underdamped cases, the mechanism is related to local minimization in $\omega_{r}^{2}-\omega^{2}$ [13], [15]. This can arise either when the resonant frequency is tuned to match the low-frequency component ω of the input signal, or when the input low-frequency component matches the resonant frequency of the system. In contrast to these earlier reports, for the acoustically excited bubble system considered here, the resonant frequency $\omega_{r}$ gets locally maximized via Ω as a result of the double-well potential condition. This follows from the fact that the resonant frequency is dependent on both *f* and Ω.

**Fig. 11:**
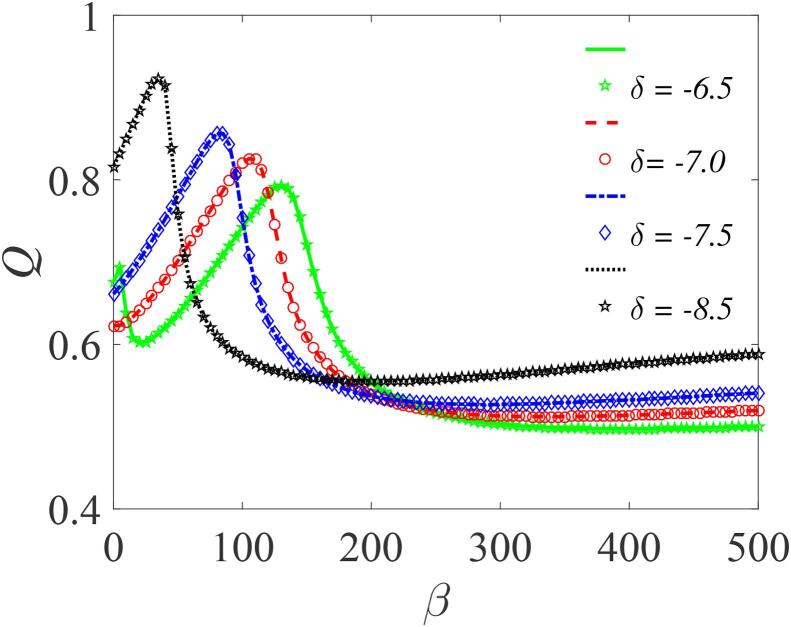
Plot of the response amplitude *Q* versus β with varying δ=-6.5,-7.0,-7.5 and -8.5; for f=10,ω=6.5,∊=0.01,λ=145,Ω=10ω and γ=-329. Continuous curves or broken lines represent the numerically-computed *Q* from Eq. (19) using Eq. (42), while the analytically calculated *Q* from Eqs. (34) and (42), are indicated by marker points.

**Fig. 12:**
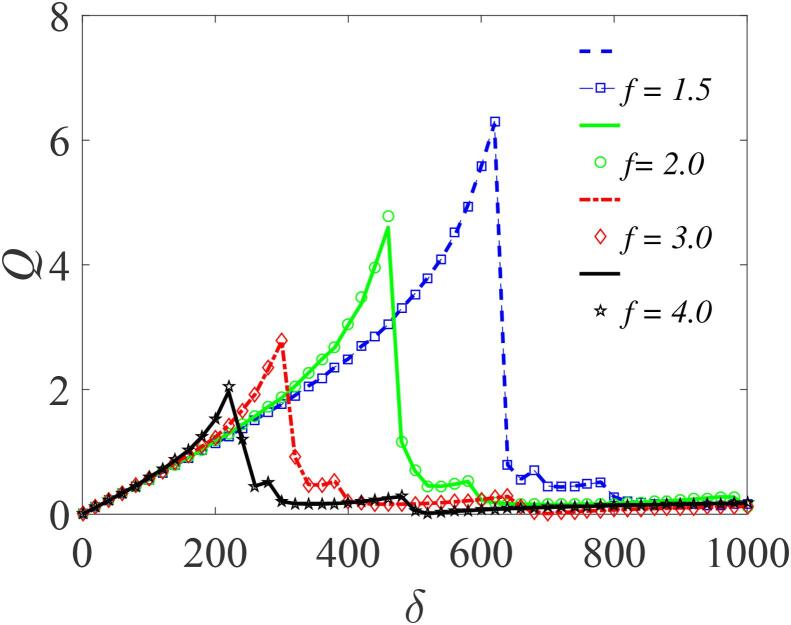
Plot of the response amplitude *Q* versus δ the effective contribution of the ambient pressure to the acoustic sound field, with by amplitude of the fast signal f=1.5,2.0,3.0 and 4.0; for Ω=30ω,ω=5,∊=0.01,β=-474,λ=145,η=1.50 and γ=-32.9. Continuous curves or broken lines represent the numerically-computed *Q* from Eq. (19) using Eq. (42), while the analytically calculated *Q* from Eqs. (34) and (42), are indicated by marker points.

To examine the effect on the response amplitude *Q* of various properties of the liquid in which the bubble oscillates, we first illustrate the dependence of *Q* on β for four different values of the liquid density related parameter δ (-6.5,-7.0,-7.5, and -8.0), as shown in Fig. 11. Here, it is clearly evident that increasing the value of δ increases the system’s response. The import is that, for constant ambient pressure $P_{0}$ and fixed equilibrium radius of the gas bubble, the bubble’s response to high-frequency modulation is strongly dependent on the liquid density. The response is large when the density of the surrounding liquid is small, and vice versa. Furthermore, in Fig. 12, the response amplitude is shown as a function of δ for different values of *f*. Evidently, at the lower values of *f* (f=1.5), the system’s response is greatly enhanced, implying that the nature and properties of the liquid can be utilized effectively in optimizing the response of the system. Although some properties of the liquid are constrained, nevertheless, in real world applications involving cavitation phenomenon, where the initial bubble radius $R_{0}$, the pressure $P_{0}$ and the fundamental frequency β are all determined by the type of application, consideration of appropriate liquids with specific properties (i.e. viscosity, surface tension and density) would provide a way of optimizing the system response.

**Fig. 13:**
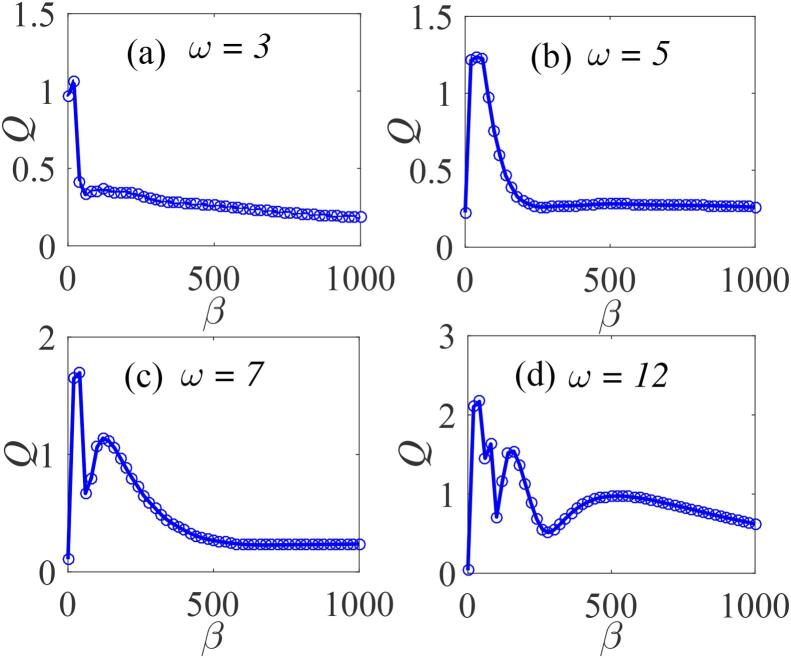
Dependence of *Q* on β for (a) ω=3 (b) ω=5 (c) ω=7 and (d) ω=12 with Ω=20ω,f=20,λ=145,∊=0.01,η=1.5,and γ=-32.9. Continuous curves represent the numerically-computed *Q* from Eq. (19) using Eq. (42), while the analytically calculated *Q* from Eqs. (34) and (42), are indicated by marker points and(or) broken lines.

To further analyse the occurrence of VR in the system subject to the double-well contitions, the dependence of *Q* on β is plotted in Fig. 13(a–d) for four values of ω, namely, ω=3,ω=5,ω=7 and ω=12 for fixed values of Ω=20ω,f=20,λ=145,∊=0.01,η=1.5 and γ=-32.9. Fig. 13(a) suggests that there is no resonance when ω is much less than 3. For ω=5 (Fig. 13(b)), the system resonates and further increase in the value of the low acoustic frequency component increases the response amplitude as well as inducing more resonance peaks. Note that this feature differs significantly from the behaviour found in the case of the single-well potential. This signifies a strong dependence of the bubble oscillator’s VR on the surface tension σ of the confining fluid medium as well as its density, ρ both of which are related to the normalized resonance frequency parameter, β. It implies that the occurrence of VR in a bubble system depends directly on parameters defining the natural frequency β.

**Fig. 14:**
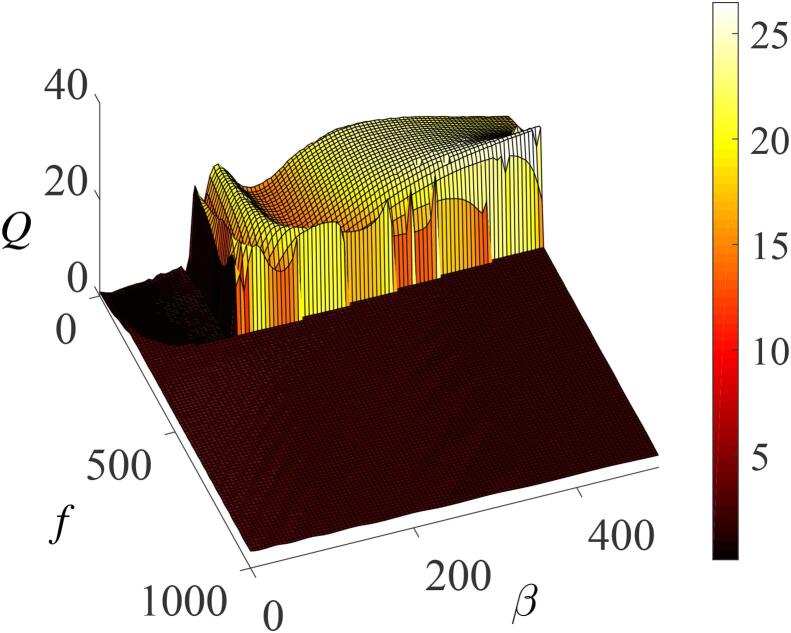
Surface plot of *Q* as a function of *f* and β for ω=20,Ω=15ω,λ=100,∊=0.01,η=1.5, and γ=400.

The curved surface of the bubble oscillator’s response amplitude *Q* as a function of the natural resonance frequency β, and the amplitude *f* of the high-frequency acoustic field, are shown in Fig. 14. In addition to the effects of the parameters of the high-frequency forcing, one can immediately observe that the effect of high-frequency acoustic field is optimized by the parameter β to enhance bubble response. This enhancement is significantly pronounced when the surface tension-dependent parameter (β) is such that β>100 and f<200. The occurrence of resonance induced by the parameter β implies that system’s response can be controlled by altering β. In practical terms, the liquid’s surface tension may be altered by adding impurities to the liquid.

**Fig. 15:**
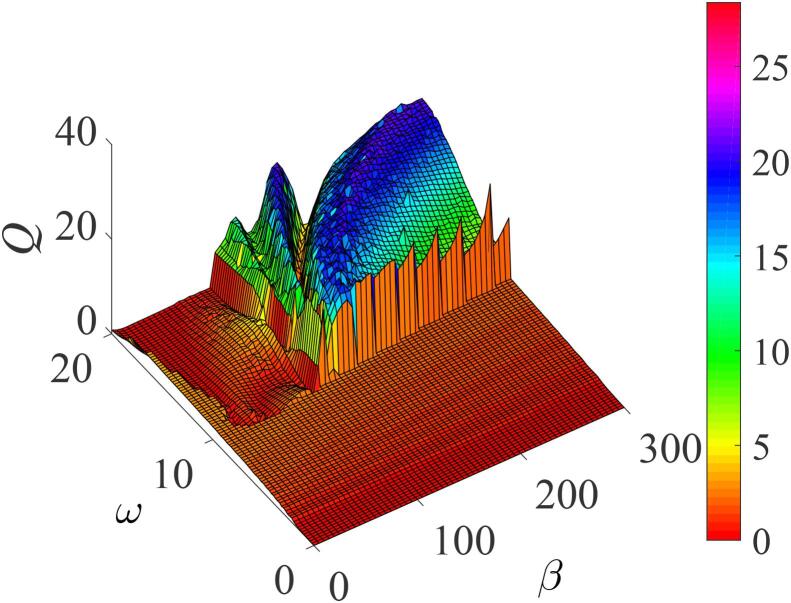
Three-dimensional plot showing the dependence of the response amplitude *Q* on the frequency of the low acoustic excitation ω and β for Ω=15ω,f=12,λ=100,δ=-12,∊=0.01,η=1.5, and γ=329.

Fig. 15, shows a three-dimensional (3D) plot of the dependence of the system’s response *Q* on the frequency of the low frequency acoustic field, ω and the natural frequency of oscillations β. Evidently, multiple resonance peaks can occur with appropriate choice of the parameter values for the high frequency acoustic driving force and, in the low frequency Ω range: Ω, with 10⩽ω⩽20. The 3D plot is characterized by two distinct and well-pronounced mountains separated by a valley wherein *Q* takes on near-zero values. While the bigger mountain occurs in the parameter regime 10<ω<20,100<β<300, the smaller mountain occurs in the neighbourhood of 15<ω<20 and 7<β<100.

As ω varies, the resonant response *Q* also varies. For ω→20, the peaks widen by increasing the spaces between them, together with a drop in the system’s maximum response. This is similar to the earlier reported vibrational resonance mechanism in a quintic Duffing oscillator with multiple potential wells [14], [15]. To complete the discussion we refer to Figs. (14) and Fig. (15) illustrating the occurrence of multi-resonance for β⩾145, which is in good agreement with our condition for the occurrence of resonance in the system obtained from the conditions imposed on the potential wells. This has earlier been established in mechanical systems where resonance behavior is dependent on the shapes of the system’s potential [8], [10], [13], [14], [15], [16], [17], [18], [19]. We remark that regimes of strong resonance mountains can also be observed even at lower values of the amplitude *f* when the response *Q* is plotted as functions of both the perturbation parameters in the range (f,Ω) ∈ [(0,100),(0,200)] with ω=15,β=400,λ=80,δ=-2,∊=0.01,η=1.5,and γ=329 satisfying the condition for the occurrence of VR in the double well-potential. In this case, the resonant frequency $\omega_{r}$ is maximized via β. The gradual increase in the values of Ω and *f* would give a wider regime of maximum response. However, when when approaching larger Ω values (Ω→20ω and higher), irrespective of the values of *f*, the resonance frequency, $\omega_{r}$ will become larger ($\omega_{r}>\omega$), giving rise to some narrow response regime for Ω>10ω, and consequently will lead to the further disappearance of the resonance peak.

## 6 Conclusion

We have examined the oscillations of an acoustically-forced gas bubble in an incompressible liquid, using the Rayleigh-Plesset bubble model. We showed that the bubble oscillation could be expressed as the dynamics of a particle in a time-dependent single or double-well potential whose properties are determined by the density of the liquid and its surface tension. The contribution of the acoustic field to the potential of the system was analysed. We considered the case of a dual-frequency acoustic driving force, consisting of a low frequency acoustic wave and a high frequency force as amplitude modulator. We investigated the occurrence of vibrational resonance (VR) numerically and validated our results theoretically, and found excellent agreement. In our analysis of VR phenomenon, both the the single- and double-well potential configurations were treated, and the conditions for the occurrence of VR obtained. For the single-well condition, the system showed a significant response on variation of the system parameter β, associated with the liquid density. In this case, single resonance peaks were observed at higher values of *f* (f⩾500) – the driving amplitude. In contrast, using the double-well potential configuration it was observed that, unlike the well-known traditional VR theory reported in the literature [8], [9], [45], [49], [136], amplification of the system’s response is achieved by varying β, instead of the parameters (*f* and Ω) of the high frequency acoustic force. This stems from the fact that the effective resonance frequency of the bubble oscillator depends on both the natural resonance frequency of the system and the parameters of the high frequency acoustic field. In addition, it was found that the appearance and disappearance of VR in the bubble system can be controlled by appropriate choice of the low frequency, ω.

Remarkably, multiple/dual-frequency bubble systems have many advantages over their single-frequency equivalent, with an abundance of practical applications as enumerated in the introduction. In contrast to numerous studies where multiple or dual-frequencies were achieved by additive application of external acoustic waves to the bubble oscillator, with the attendant need for large ancillary equipment to provide the external fields, dual-frequencies can be achieved instead through amplitude modulation of single-frequency acoustic waves. The acoustic vibrations of the bubble oscillator demonstrated here can be exploited to facilitate the nucleation of bubbles in biological tissues, enhancing the bubble growth rate as well as generating extremely violent forces during bubble collapse. The growth processes occur during cavitation and can be controlled by the parameters defining the properties (e.g. viscosity and surface tension) of the medium in which the bubble oscillates and the characteristics of the applied ultrasound field (amplitude and frequency). Specifically, control of the applied ultrasound energy can be achieved through the dynamic multifrequency radiation force usually created using ultrasound equipped with time-dependent high-frequency periodic amplitude modulation [137]. Thus, our simulated results could have direct applications to the production of polymer foams and to the ultrasonic degassing of liquids, as well as to the fabrication of porous ceramics – all of which undergo bubble nucleation events. Indeed, an amplitude modulation mechanism has been experimentally demonstrated recently for efficient enhancement of acoustically-driven micromixing and microcentrifuging processes, which has the advantage of eliminating the requirement for large ancillary equipment necessary for the provision of multiple external fields [138]. Finally, in biological applications, the parameters of the ultrasonic fields can be adjusted selectively to ensure that damage to larger vessels is significantly reduced or to favour the retention of extracellular matrix;, which can be achieved by modulating the amplitude of the acoustic wave using high frequency modulating forces.

## CRediT authorship contribution statement

**K.A. Omoteso:** Methodology, Visualization, Data curation, Software, Validation, Investigation, Formal analysis, Writing - original draft. **T.O. Roy-Layinde:** Data curation, Visualization, Investigation, Software, Validation, Formal analysis, Writing - original draft. **J.A. Laoye:** Software, Validation, Formal analysis, Project administration, Supervision, Writing - original draft, Writing - review & editing. **U.E. Vincent:** Conceptualization, Methodology, Investigation, Software, Formal analysis, Resources, Project administration, Supervision, Writing - original draft, Writing - review & editing. **P.V. E. McClintock:** Funding acquisition, Project administration, Resources, Supervision, Writing - review & editing.

## Declaration of Competing Interest

The authors declare that they have no known competing financial interests or personal relationships that could have appeared to influence the work reported in this paper.

## Appendix A Appendix – fixed points and stability of equilibria

### A.1 Appendix I

The fixed points $\left(x_{*},y_{*}\right)$ of the autonomous bubble system are obtained by setting

(46) y=0,

(47) $$\beta x-\gamma x^{2}+\gamma x^{3}-\lambda x^{4}=0\text{.}$$

By expressing Eq. (47) as $x(\beta -\gamma x+\gamma x^{2}-\lambda x^{3})=0$, then, x=0 is a solution and always exists. Thus, $x_{0}=0$ always exists and is a trivial fixed point of the system such that $P_{1}(0,0)$ is an equilibrium. The other three fixed points can be obtained by solving $(\beta -\gamma x+\gamma x^{2}-\lambda x^{3})=0$, which gives $x_{\beta }=\beta$ as the second fixed point that always exists when β≠0. The corresponding equilibrium point to $x_{\beta }$ is denoted by $P_{2}(\beta ,0)$. Finally, the last two fixed points are obtained from the auxiliary condition

$$\lambda x^{2}-\gamma x+(\gamma -\beta )=0,$$

which gives $x_{\pm }=\frac{\gamma \pm \sqrt{\Delta }}{2\lambda }$ with the discriminant $\Delta =\gamma^{2}-4\lambda (\gamma -\beta )$. The nature of the fixed points $x_{\pm }$ is determined by the sign of β and can be examined for β<0 and β>0.

A. When β<0, there are six possible cases.
  Case (i): β<0,γ=λ=0, then, $x_{\pm }=0$ exist and corresponds to the trivial fixed point $x_{0}$.
  Case (ii): β<0,γ=0,λ>0, then, $x_{\pm }=\frac{\pm i\sqrt{4\lambda \beta }}{2\lambda }$ exist and are complex.
  Case (iii): β<0,γ>0λ>0, then,
  $x_{\pm }=\frac{\gamma \pm \sqrt{\Delta_{1}}}{2\lambda },\Delta_{1}=\gamma^{2}-4\lambda (\gamma +\beta )$. This implies that(a) If $\Delta_{1}>0$, then, $x_{\pm }$ exist and are real.(b) If $\Delta_{1}<0$, then, $x_{\pm }$ exist and are complex.
  Case (iv): β<0,γ>0λ<0, then, $x_{\pm }=\frac{-\gamma \pm \sqrt{\Delta_{2}}}{2\lambda }$, and $x_{\pm }$ are real and always exist where $\Delta_{2}=\gamma^{2}+4\lambda (\gamma +\beta )$.
  Case (v): β<0,γ<0λ>0, then, $x_{\pm }=\frac{-\gamma \pm \sqrt{\Delta_{2}}}{2\lambda }$, and $x_{\pm }$ are real and always exist.
  Case (vi): β<0,γ<0λ<0, then, $x_{\pm }=\frac{-\gamma \pm \sqrt{\gamma^{2}+4\lambda (\beta -\gamma )}}{2\lambda }$. This implies that $x_{\pm }$ exist with one real and one complex root whenever |β|<|γ| and $\gamma^{2}<4\lambda (\beta -\gamma )$. This depends on the values of the parameters.
B. When β>0, there are also six possible cases.
  Case (i): β>0,γ=λ=0, then, $x_{\pm }=0$ exist, corresponding to the trivial fixed point $x_{0}$.
  Case (ii): β>0,γ=0,λ>0. Then, $x_{\pm }=\frac{\pm i\sqrt{4\lambda \beta }}{2\lambda }$, exist and are complex.
  Case (iii): β>0,γ=0,λ<0. then, $x_{\pm }=\frac{\mp i\sqrt{4\lambda \beta }}{2\lambda }$ exist and are complex as in case (iii).
  Case (iv): β>0,γ<0λ>0, then, $x_{\pm }=\frac{-\gamma \pm \sqrt{\Delta_{2}}}{2\lambda }$, implying that $x_{\pm }$ always exist and are real.
  Case (v): β>0,γ>0λ>0, then, $x_{\pm }=\frac{\gamma \pm \sqrt{\Delta_{3}}}{2\lambda },\Delta_{3}=\gamma^{2}-4\lambda (\gamma -\beta )$. The existence of $x_{\pm }$ depends on the absolute |λ| and |γ| relative to β. This implies that,(a) if $\Delta_{3}>0$, then, $x_{\pm }$ are real.(b) if $\Delta_{3}<0$, then, $x_{\pm }$ exist and consists of one real and one complex.
  Case (vi): β>0,γ>0λ<0, then, $x_{\pm }=\frac{-\gamma \pm \sqrt{\Delta_{4}}}{2\lambda },\Delta_{4}=\gamma^{2}+4\lambda (\gamma -\beta )$ implying that,(a) if (γ-β)>0, then, $x_{\pm }$are real.(b) if (γ-β)<0, then, $x_{\pm }=\frac{-\gamma \pm \sqrt{\gamma^{2}-4\lambda P_{-}}}{2\lambda }$with $P_{-}=|(\gamma -\beta )|$.
  ⇒ (i) If $\gamma^{2}-4\lambda P_{-}>0$, then, $x_{\pm }$ exist and are real.
  ⇒ (ii). If $\gamma^{2}-4\lambda P_{-}<0$, then, $x_{\pm }$ exist and consists of one real and one complex.

In summary, it follows from A and B that $x_{\pm }=\frac{\gamma \pm \sqrt{\Delta }}{2\lambda }$ are two non-trivial fixed points and the two solutions $x_{+}$ and $x_{-}$ corresponds to two equilibrium points $P_{3,4}$($\frac{\gamma \pm \sqrt{\Delta }}{2\lambda },0$).

Thus, the unperturbed system has four equilibrium points:

$$P_{1}(0,0),P_{2}(\beta ,0),P_{3}\left(\frac{\gamma +\sqrt{\Delta }}{2\lambda },0\right),P_{4}\left(\frac{\gamma -\sqrt{\Delta }}{2\lambda },0\right)\text{.}$$

Remarks 6.1 The equilibrium point,

(i) $P_{1}(0,0)$ is trivial.
(ii) $P_{2}(\beta ,0)$ is unique.
(iii) $P_{3}\left(\frac{\gamma +\sqrt{\Delta }}{2\lambda },0\right)$ is non-trivial.
(iv) $P_{3}\left(\frac{\gamma -\sqrt{\Delta }}{2\lambda },0\right)$ is non-trivial.

To examine the stability of the equilibrium points, we write the Jacobian matrix at the equilibrium points $P_{i}(x_{*},0)$, where $x_{*}$ are the fixed points, as

(48) $$J=\left(\begin{array}{cc} 0 & 1\\ \beta -2\gamma x_{*}+3\gamma x_{*}^{2}-4\lambda x_{*}^{3} & 0 \end{array}\right)$$

1. The Jacobian matrix for the trivial equilibrium point $P_{1}(0,0)$ is

(49) $$J_{0}=\left(\begin{array}{cc} 0 & 1\\ \beta & 0 \end{array}\right)\text{.}$$

The characteristic equation is $\lambda^{*2}-\beta =0$ and the eigenvalues are $\lambda_{\pm }^{*}=\pm \sqrt{\beta }$.

(i) If $\beta >0,\Leftrightarrow \lambda_{+}^{*}\&\lambda_{-}^{*}$ are real with opposite signs and then the fixed point $P_{1}$ is a saddle node.
(ii) If $\beta <0,\Leftrightarrow \lambda_{\pm }^{*}=\pm i\sqrt{\beta }$, such that $\lambda_{+}^{*}\&\lambda_{-}^{*}$ are complex and the fixed point $P_{1}$ is a centre.

2. The Jacobian matrix for the unique equilibrium point $P_{2}(\beta ,0)$ is,

(50) $$J_{P_{2}}=\left(\begin{array}{cc} 0 & 1\\ \beta -2\gamma \beta +3\gamma \beta^{2}-4\lambda \beta^{3} & 0 \end{array}\right)\text{.}$$

The characteristic equation is $\lambda^{*2}-\zeta =0$ with $\zeta =\beta -2\gamma \beta +3\gamma \beta^{2}-4\lambda \beta^{3}$. The eigenvalues are $\lambda_{\pm }^{*}=\pm \sqrt{\zeta }$.

(i) If $\zeta >0,\Leftrightarrow \lambda_{+}^{*}\&\lambda_{-}^{*}$ are real with opposite signs and the fixed point $P_{2}$ is a saddle node.
(ii) If $\zeta <0,\Leftrightarrow \lambda_{\pm }^{*}=\pm i\sqrt{\zeta }$, then $\lambda_{+}^{*}\&\lambda_{-}^{*}$ are complex and the fixed point $P_{2}$ is a centre.

3. The Jacobian matrix for the non-trivial equilibrium point $P_{3}\left(\frac{\gamma +\sqrt{\Delta }}{2\lambda },0\right)$ is,

(51) $$J_{P_{3}}={\left(\begin{array}{cc} 0 & 1\\ \beta -2\gamma x_{*}+3\gamma x_{*}^{2}-4\lambda x_{*}^{3} & 0 \end{array}\right)}_{x_{*}=P_{3}}\text{.}$$

The characteristic equation is $\lambda^{*2}-\varpi =0$, with

$$\varpi =\beta -2\gamma \left(\frac{\gamma +\sqrt{\Delta }}{2\lambda }\right)+3\gamma {\left(\frac{\gamma +\sqrt{\Delta }}{2\lambda }\right)}^{2}-4\lambda {\left(\frac{\gamma +\sqrt{\Delta }}{2\lambda }\right)}^{3}\text{.}$$

The eigenvalues are $\lambda_{\pm }^{*}=\pm \sqrt{\varpi }$, and two cases arise:

(i) If $\varpi >0,\Leftrightarrow \lambda_{+}^{*}\&\lambda_{-}^{*}$ are real with opposite signs and the fixed point $P_{3}$ is a saddle node.
(ii) If $\varpi <0,\Leftrightarrow \lambda_{\pm }^{*}=\pm i\sqrt{\varpi }$, then $\lambda_{+}^{*}\&\lambda_{-}^{*}$ are complex and the fixed point $P_{3}$ is a centre.

4. Equilibrium point $P_{4}\left(\frac{\gamma -\sqrt{\Delta }}{2\lambda },0\right)$.

(52) $$J_{P_{4}}={\left(\begin{array}{cc} 0 & 1\\ \beta -2\gamma x_{*}+3\gamma x_{*}^{2}-4\lambda x_{*}^{3} & 0 \end{array}\right)}_{x_{*}=P_{4}}\text{.}$$

The characteristic equation is $\lambda^{*2}-\xi =0$ with

$$\xi =\beta -2\gamma \left(\frac{\gamma -\sqrt{\Delta }}{2\lambda }\right)+3\gamma {\left(\frac{\gamma -\sqrt{\Delta }}{2\lambda }\right)}^{2}-4\lambda {\left(\frac{\gamma -\sqrt{\Delta }}{2\lambda }\right)}^{3}\text{.}$$

The eigenvalues are $\lambda_{\pm }^{*}=\pm \sqrt{\xi }$,

(i) If $\xi >0,\Leftrightarrow \lambda_{+}^{*}\&\;\lambda_{-}^{*}$ are real with opposite signs and the fixed point $P_{3}$ is a saddle node.
(ii) If $\xi <0,\Leftrightarrow \lambda_{\pm }^{*}=\pm i\sqrt{\xi }$, then $\lambda_{+}^{*}\&\lambda_{-}^{*}$ are complex and the fixed point $P_{4}$ is a centre.

### A.2 Appendix II

Here, we present the analytical results for the fixed points in terms of $\chi^{*}$ by equating the effective potential $V_{\mathrm{eff}}(\chi )$ given by Eq. (39) to zero. This, yields five fixed points solutions for $\chi^{*}$ given as:

$$\begin{array}{cc} \chi_{1}^{*}= & 0,\\ \chi_{2,3}^{*}= & -\frac{\gamma }{4\lambda }-\frac{1}{2}\sqrt{\frac{\frac{\gamma^{2}}{4\lambda^{2}}+\frac{2C_{5}}{3\lambda }+2^{1/3}\Gamma }{\left[3\lambda +\frac{1}{3(2^{1/3})\lambda }\right]\Xi }}\\ & \pm \frac{1}{2}\sqrt{\frac{\frac{\gamma^{2}}{2\lambda^{2}}+\frac{4C_{5}}{3\lambda }-2^{1/3}\Gamma }{\left[3\lambda -\frac{1}{3(2^{1/3})\lambda }\right]\Xi }}-\left(\frac{-\frac{\gamma^{3}}{\lambda^{3}}-\frac{4\gamma C_{5}}{\lambda^{2}}-\frac{8C_{4}}{\lambda }}{4\sqrt{\frac{\frac{\gamma^{2}}{4\lambda^{2}}+\frac{2C_{5}}{3\lambda }+2^{1/3}\Gamma }{\left[3\lambda +\frac{1}{3(2^{1/3})\lambda }\right]\Xi }}}\right),\\ \chi_{4,5}^{*}= & -\frac{\gamma }{4\lambda }+\frac{1}{2}\sqrt{\frac{\frac{\gamma^{2}}{4\lambda^{2}}+\frac{2C_{5}}{3\lambda }+2^{1/3}\Gamma }{\left[3\lambda +\frac{1}{3(2^{1/3})\lambda }\right]\Xi }}\\ & \pm \frac{1}{2}\sqrt{\frac{\frac{\gamma^{2}}{2\lambda^{2}}+\frac{4C_{5}}{3\lambda }-2^{1/3}\Gamma }{\left[3\lambda -\frac{1}{3(2^{1/3})\lambda }\right]\Xi }}+\left(\frac{-\frac{\gamma^{3}}{\lambda^{3}}-\frac{4\gamma C_{5}}{\lambda^{2}}-\frac{8C_{4}}{\lambda }}{4\sqrt{\frac{\frac{\gamma^{2}}{4\lambda^{2}}+\frac{2C_{5}}{3\lambda }+2^{1/3}\Gamma }{\left[3\lambda +\frac{1}{3(2^{1/3})\lambda }\right]\Xi }}}\right)\text{.} \end{array}$$

Where $\Gamma ,\Xi ,\Delta_{1},\Delta_{2}$ and $\Delta_{3}$ are defined as,

$$\begin{array}{cc} \Gamma = & C_{5}^{2}+3C_{4}\gamma -12C_{6}\lambda ,\\ \Xi = & {\left(-2C_{5}^{3}+9C_{4}C_{5}\gamma -27C_{6}\gamma^{2}+\Delta_{1}+\sqrt{\Delta_{3}}\right)}^{1/3},\\ \Delta_{1}= & 27C_{4}^{2}\lambda -72C_{5}C_{6}\lambda ,\\ \Delta_{2}= & 27C_{6}\gamma^{2}+27C_{4}^{2}\lambda ,\\ \Delta_{3}= & -4\Gamma^{3}+{\left(-2C_{5}^{3}+9C_{4}C_{5}\gamma -\Delta_{2}-72C_{5}C_{6}\lambda \right)}^{2}, \end{array}$$

indicating the concurrences of our numerical and analytical results.
